# The importance of genotyping within the climate-smart plant breeding value chain – integrative tools for genetic enhancement programs

**DOI:** 10.3389/fpls.2024.1518123

**Published:** 2025-02-06

**Authors:** Ana Luísa Garcia-Oliveira, Rodomiro Ortiz, Fatma Sarsu, Søren K. Rasmussen, Paterne Agre, Asrat Asfaw, Moctar Kante, Subhash Chander

**Affiliations:** ^1^ Genetic Resources Program, Alliance Bioversity International and International Center for Tropical Agriculture (CIAT), Cali, Colombia; ^2^ Department of Plant Breeding, Swedish University of Agricultural Sciences, Alnarp, Sweden; ^3^ Plant Breeding and Genetics Section, Joint FAO/IAEA Center, International Atomic Energy Agency, Vienna, Austria; ^4^ Independent Researcher, Roskilde, Denmark; ^5^ Yam Breeding Unit, International Institute of Tropical Agriculture, Ibadan, Nigeria; ^6^ Genetics, Genomics, and Crop Improvement Division, International Potato Center, Lima, Peru; ^7^ Oilseeds Section, Department of Genetics & Plant Breeding, CCS Haryana Agricultural University, Hisar, India

**Keywords:** agriculture, marker genotyping, mutation breeding, NGT, CRISPR, legislation, climate-smart cultivars

## Abstract

The challenges faced by today’s agronomists, plant breeders, and their managers encompass adapting sustainably to climate variability while working with limited budgets. Besides, managers are dealing with a multitude of issues with different organizations working on similar initiatives and projects, leading to a lack of a sustainable impact on smallholder farmers. To transform the current food systems as a more sustainable and resilient model efficient solutions are needed to deliver and convey results. Challenges such as logistics, labour, infrastructure, and equity, must be addressed alongside adapting to increasingly unstable climate conditions which affect the life cycle of transboundary pathogens and pests. In this context, transforming food systems go far beyond just farmers and plant breeders and it requires substantial contributions from industry, global finances, transportation, energy, education, and country developmental sectors including legislators. As a result, a holistic approach is essential for achieving sustainable and resilient food systems to sustain a global population anticipated to reach 9.7 billion by 2050 and 11.2 billion by 2100. As of 2021, nearly 193 million individuals were affected by food insecurity, 40 million more than in 2020. Meanwhile, the digital world is rapidly advancing with the digital economy estimated at about 20% of the global gross domestic product, suggesting that digital technologies are increasingly accessible even in areas affected by food insecurity. Leveraging these technologies can facilitate the development of climate-smart cultivars that adapt effectively to climate variation, meet consumer preferences, and address human and livestock nutritional needs. Most economically important traits in crops are controlled by multiple loci often with recessive alleles. Considering particularly Africa, this continent has several agro-climatic zones, hence crops need to be adapted to these. Therefore, targeting specific loci using modern tools offers a precise and efficient approach. This review article aims to address how these new technologies can provide a better support to smallholder farmers.

## 1 Addressing food crisis sustainability under the current climatic challenges

According to the FAO et al. (2019), over 820 million people globally live with hunger, which is a phenomenon rising in almost all subregions of Africa, and to a lesser extent in Latin America and Asia. Since 2014, food insecurity across the sub-Saharan Africa region, particularly South Sudan and Nigeria, has been exacerbated by human conflict as well as severe and long periods characterized by drought and sudden pests’ attacks, particularly affecting pastoralists (Anderson et al., 2021). Drought is known to be one of the main factors behind the undernourishment increase in this region (FAO et al., 2019) and has been consistently identified as a primary driver of famines (Maxwell and Hailey, 2020), especially in countries such as Ethiopia, Nigeria, and South Sudan.

**Figure 1:**
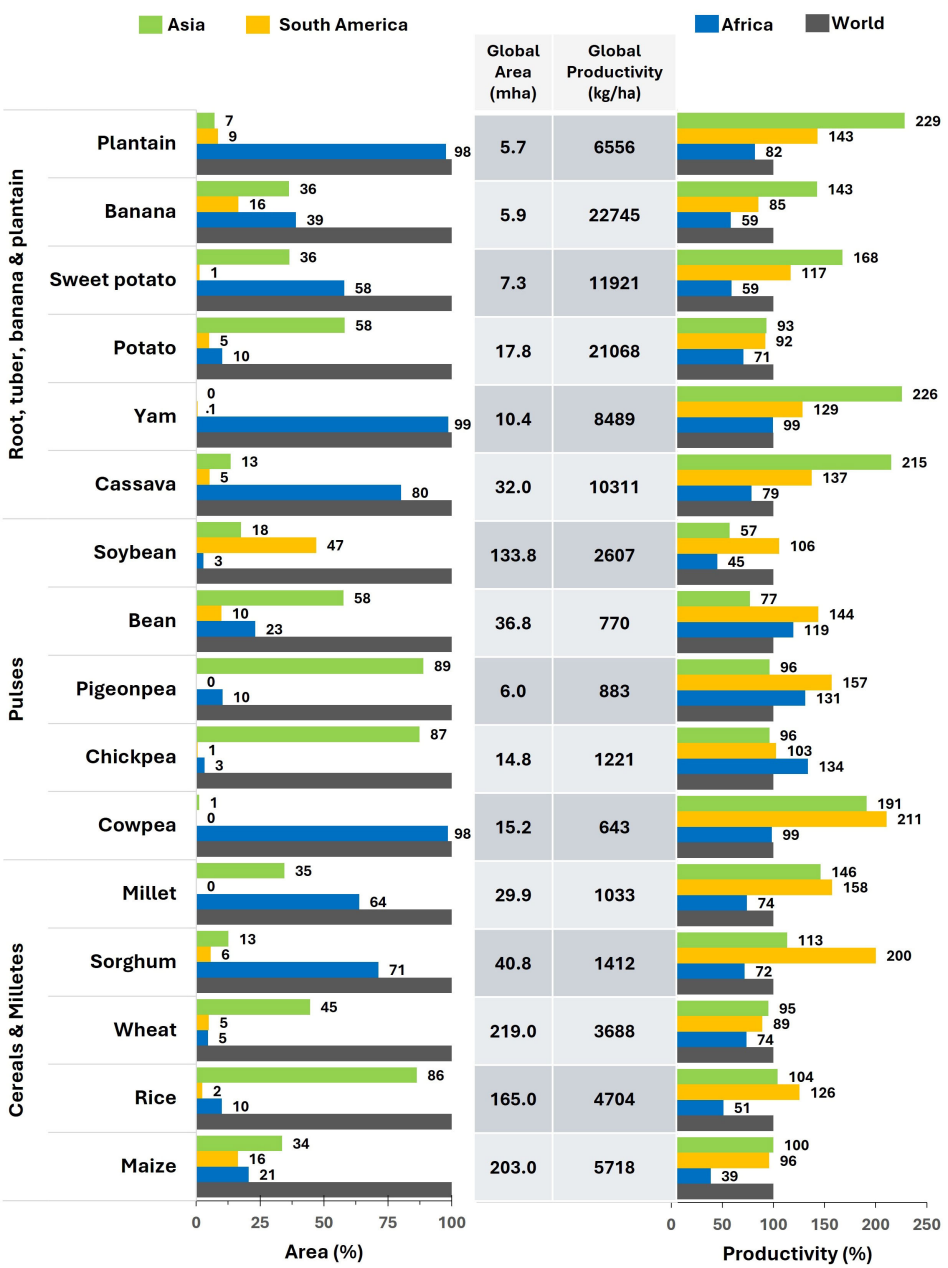
Major field crop area *vs.* productivity in Africa, Asia, South America and world. The figure illustrates that in Africa, yam, cassava, plantain, millets and sorghum are crucial crops for maintaining food security sustaining the need for more breeding investments in these crops. Pigeonpeas, chickpeas, common beans, potato, but also maize, rice and wheat are examples of crops in which productivity exceeds the cultivated area. (Source: FAODATA, 2022).

Yet, alongside insufficient food intake, there is a contrasting issue where both children and adults suffer from obesity, diabetes, and other food-related diseases due to the consumption of low-quality and nutritionally poor food. In fact, obesity, along with overweight, contributes to approximately four million deaths annually worldwide. The connection between obesity and food insecurity is partly driven by high food costs and the widespread reliance on inexpensive sources of fats and sugars (GBD 2015 Obesity Collaborators et al., 2017). Despite the apparent stabilization in the global hunger trend observed throughout the 2021–2022 period, the concern remains regarding the significant number of women in rural areas who continue to struggle with accessing nutritious, safe, and sufficient food (FAO, 2023). Hunger remains a persistent challenge in least-developed regions, especially across all sub-regions of Africa (FAO, 2023) because substantial gaps between potential and actual crop yields continue to hinder food security (**Figure 1**). Extreme weather, particularly variation on temperature and rainfall, along with limited resource availability, further contribute to disparities in crop production across the different regions. For example, rainfed upland rice systems are more sensitive to soil moisture variability when compared to irrigated paddy rice systems (Stuecker et al., 2018). Thus, selecting cultivars that meet needs and expectations while providing stable incomes is quite challenging. The impact of heat and drought on crops varies by season and geography, with evidence showing that inter-annual climate variability can significantly affect harvested yields and overall production (Roberts et al., 2009; Lizumi and Ramankutty, 2016). Only climate-smart plant breeding can provide new cultivars that are tolerant to heat and drought.

Recent findings from the Agricultural Model Intercomparison and Improvement Project (AgMIP), and Inter-Sectoral Impact Model Intercomparison Project (ISIMIP) showed that higher temperatures generally result in lower grain yields for maize, soybean, and rice. Conversely wheat yields have been found to increase due to higher CO_2_ concentrations together with expanded high latitude regions (Jägermeyr et al., 2021). In the Iberian Peninsula, spring maximum temperatures contribute to significant grain yield losses in barley and rainfed wheat, but also for reducing the marketable tuber production in potatoes. However, northern regions are projected to experience increased grain yields due to early winter warming which promotes earlier growth of seedlings (Bento et al., 2021). These changes suggest that adjustments in agricultural practices, management and selection of crop species and cultivars are necessary.

## 2 Cause root and derived actions

The Paris Declaration endorsed the base development efforts on first-hand experience rather than simply conveying aid. It was supported by five main pillars: ownership, alignment, harmonization, result management, and accountability. However, despite these well-intentioned principles, the desire for quick results may have contributed to a lack of clarity and coordination in the development process, leading to incomplete fulfilment of providing nutritious and healthy food for all in a sustainable manner though current food systems. The impediments are many and may lie beyond ‘agricultural’ restrictions. Imbalances related to internal governance, either from the economic or territorial sides, do not convey the fairness of expected returns. Under a set of partnerships between the European Union (EU), the Food and Agriculture Organization of the United Nations (FAO/UN), the French Agricultural Research Centre for International Development (CIRAD), and national stakeholders, key points, including food security, nutrition and health were gathered for inclusive and sustainable solutions to transform food systems while preserving ecosystems and landscapes (FAO, 2021).

Africa, with its 55 countries, is the continent with the highest number of countries, and the highest population growth rate. By 2100, five of the 10 top-most populous countries in the world will be in Africa, accounting for over half of the projected global population growth through 2050. Additionally, the problem of inadequate access to food in countries affected by conflict is worsened by natural disasters, economic challenges, and public health issues (UN-DESA, 2022). This means that in Africa there is a high demand for increased productivity (**Figure 1**), and to counteract rising food prices, annual production needs to grow substantially. In these endowers should be acknowledged multiple efforts made in water management and some plant varieties adapted to low input conditions (Nyika and Dinka, 2023; CIMMYT, 2021).

**Figure 2:**
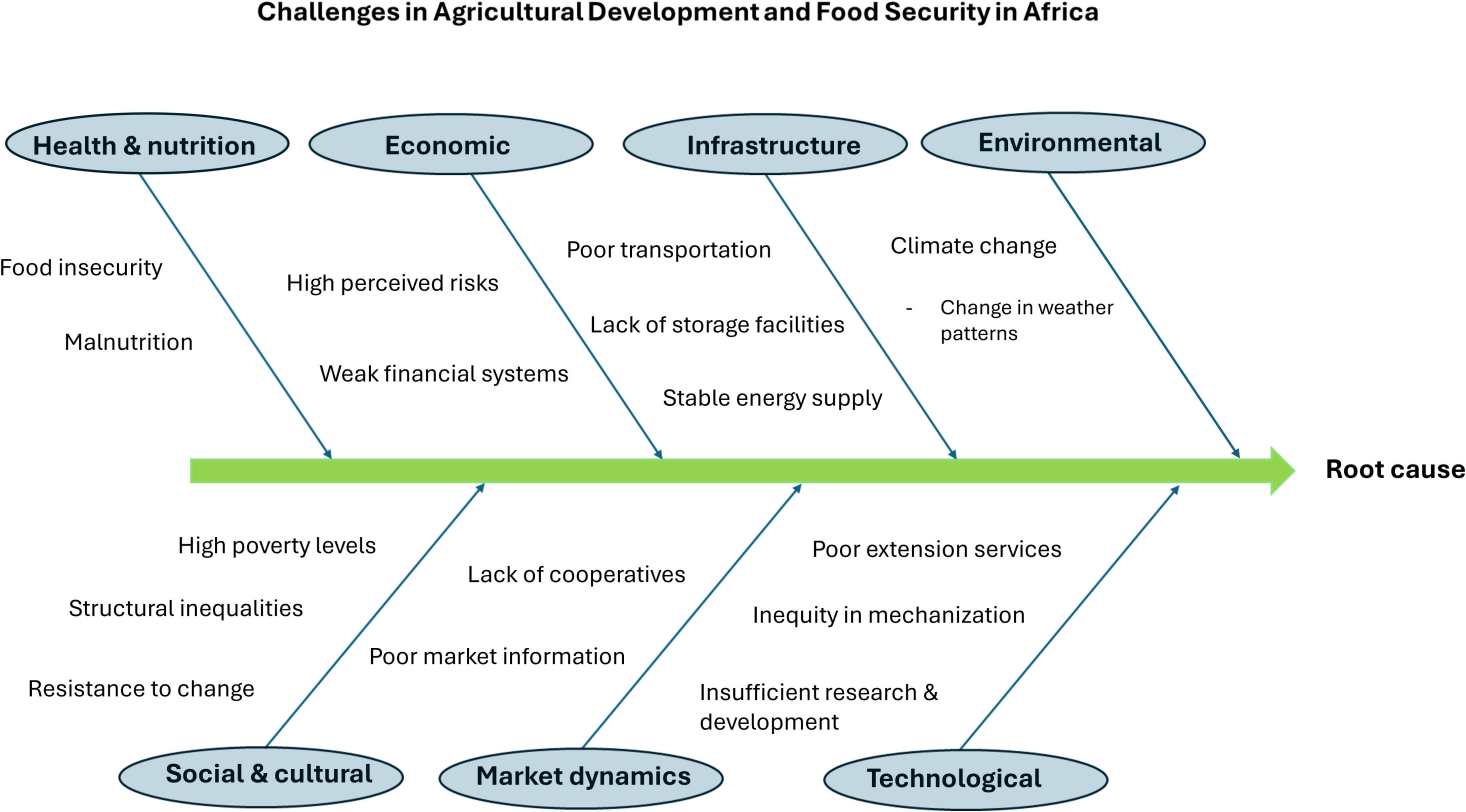
Root cause analysis of the challenges faced by the African continent in agricultural development. Additional issues stated in the backbone diagram include emphasis in quick results, confusion among the stakeholders particularly when not involving the local communities in decision making and setting priorities, as well conflicts leading to lack of trust among partners.

Criticism was raised when it was pointed out that major efforts to improve water and nutrient efficiency in plants have not produced major results in the tropics, ignoring the fact that the advantages of crop improvement research and social protection programs are relative and dependent on the situation in a particular region (McIntire and Dobermann, 2023; Ginkel and Cherfas, 2023) (**Figure 2**).

In climate-smart plant breeding, the tackling of these challenges involves separate genetic selection for rainfed and irrigated conditions and for resistance to pests and pathogens. Additional actions include research efforts aimed at rejuvenating growth in crops that have reached a plateau yield improvement.

Besides the use of hybrid cultivars, the increase in grain yield in crops is known to be positively correlated with increased levels of nitrogen (N) fertilizers (Maheswari et al., 2017). Yet, despite N-fertilizers being a key determinant of yield, it also represents a significant cost for the farmers and in addition may have a negative environmental impact. An example is nitrate leaching from the field leading to soil and underground water contaminations as well as to greenhouse gas emissions. In sub-Saharan Africa, transformation in productivity can be achieved quickly through increased use of fertilizers and irrigation management. Yet, it is essential to consider that applying fertilizers without irrigation management has limited benefits, and the cost of irrigation can be a limiting factor (McIntire and Dobermann, 2023). Actions in this direction include breeding for nitrogen use efficiency (NUE: grain dry matter yield *per* unit of N availability) as well as tentative reduction of excess N-fertilizer input by management while maintaining an acceptable yield and quality. NUE is particularly important for smallholder farmers since they are known to have few resources at their disposal at any given time with which they work under very stringent financial constraints. Hence, this means that farmers cannot afford the luxury of buying large quantity of fertilizers as is always the case where one system is used for both feeding people and generating income. The trait NUE is controlled by many genes and fortunately many of these have in the recent years been genotyped and amenable for employing integrative tools for genetic enhancement of crop plants.

Optimization within breeding programs (BPs) is being led by management and optimization of the BP itself, using standardized tools and services. Yet, this may not necessarily lead to the desired improvements, as plant breeding is currently limited by the reliance on the selection of rare naturally occurring mutations in gene-regulatory regions and for traits that are controlled by multiple genes (Dwivedi et al., 2023). Rare alleles are mostly under-looked in the majority of quantitative genetic analyses since these are found in very few individuals of a population (Garcia-Oliveira et al., 2014), e.g. within less than a 2% frequency in a given population of a crop such as rice (Purugganan and Jackson, 2021).

Having a diverse set of alleles related to nitrogen uptake, assimilation, and utilization can contribute to improved NUE in plants. These alleles may also carry specific traits that make plants more adaptive to nitrogen-limited conditions. For example, in bread wheat, certain cultivars are more prone to aluminium stress because a panoply of physiological traits making plants more resistant to the damages incurred by aluminium in the soil (Garcia-Oliveira et al., 2016). In the case of N, the rare allele might encode a transporter protein with a higher affinity for nitrogen uptake from the soil, allowing the plant to acquire and utilize nitrogen more efficiently. If such rare alleles are identified and successfully incorporated in the target crop through breeding, it could result in a cultivar with improved NUE. Recently, Yoon et al. (2022) discovered that the nonsense-mutated *GS3* gene, represented by the *gs3* allele in the rice cultivar ‘Akita 63’, enhances yield production and grain size in rice, thereby improving harvest index and NUE in rice. With the newest mutational breeding tools the discovery can be transferred to other crop species swiftly.

## 3 Climate-smart cultivar selection

When it comes to cultivar substitution and recommendation, it is important to consider inter-variability periodicity. Adjustment to identify main parameters including genotype nature (G), environment (E), and their interaction (G×E) would improve the predictions. The main aim is to make correct forecast and decisions that would result in maximum outcomes. Besides, the ploidy level, genotypic value, and allele frequency must also be taken into consideration. When considering G×E interaction parameters related to biotic and abiotic stress tolerance, a mean performance across different trials conducted under various field management (M), effects of the trial (E), repetition (e), genotype (G), interaction of genotype k and trial i (G_k_E_i_), residual error, number of trials and number of repetitions per trial should be taken into consideration in the model applied. Plant breeders focus on specific traits, also known as TPTs (*T*arget *P*erformance *T*raits), when developing new cultivars with improved tolerance, for example to abiotic stresses. Defining these traits, which form the basis for the ideotype concept (Donald, 1968), is crucial for effectively selecting and breeding plants that can withstand challenging environmental conditions. With reference to African climate and conditions, the G×E effects may be hugely underestimated for operation efficacy, along with a lack of resources. More precisely, four major agro-climatic zones, can be considered, and each characterized by distinct climate, soil, and vegetation types, namely the a) Arid and Semi-Arid Zone with low rainfall and high temperature (e.g. Sudano-Sahelian zone), the b) Sub-Humid Zone where the rainfall frequency is more reliable (e.g. coastal areas), the c) Humid zone characterized by high rainfall and supporting lush vegetation and diverse crops (e.g. the Central and southern parts of West Africa), and finally the d) Tropical high-altitude zone, typically comprised of highland areas characterized by cooler temperatures and distinct growing seasons (e.g. East African highlands of Ethiopia and Kenya as well the Rift valley of Tanzania). Studying these interactions may be very demanding, but principal component analysis (PCA) may help in this multifactorial-dependent process. Taken this continent as an example breeder’s face challenges specific to each climate zone where water scarcity, pests and disease pressure, and soil problems vary within these zones. Developing climate-smart cultivars, such as drought-resistant varieties for arid and semi-arid zones, involves more than just adaptation to a changing environment. It must first prioritize customization to the consumer’s needs and preferences. These market choices drive producers to use and harvest cultivars fitted to the unfavourable environment due to exposure to erratic edaphoclimatic conditions in terms of frequency and intensity (**Table 1**). For cultivar selection a significant genetic variation is needed, and where germplasm diversity is met by the high phenotypic variability provided by wild relatives, old cultivars, and landraces into elite lines with a known and stable yield production background (Garcia-Oliveira et al., 2009). Because climatic conditions evolve, cultivar replacement is thought to be a more frequent need when the primary focus is on supply-driven concerns. More focus should be put on adding locations (multi-site trials) as opposed to years (multi-year trials) (van Etten et al., 2019), through farmer-participatory on-farm trials. The advantages to this approach include that location variables tend to be more predictable than year variables, and one could target locations with different variable profiles to get well-adapted cultivars. Additionally, it attracts a greater number of participants and affords grower cooperators a meaningful presence in the discussions or decision-making processes. Yet, decisions from the breeding side must take into consideration their resource allocation of what is feasible in terms of number of years, number of sites, and how to choose sites to maximize adaption/climate-smartness.

**Table 1:** Predicted impact on global agricultural potential based on the projected trends in climatic change scenarios and its management through mitigation/adaptation practices.

Factor	Event	Potential impact	Likelihood	Mitigation/adaptation Practices
Temperature	Cold periods becoming warmer and shorter; over most land areas, days and nights becoming hotter.	Positive impact on crop yields in cooler environments particularly northern hemisphere countries, but it is likely to be either offset or severe reduction in warmer environments (tropical and sub-tropical), increased outbreaks of new insect pests and pathogens resulting in potential impacts on crop production; change in the life cycle of bees, and as consequence, in pollination and plant fertility.	Virtually certain	Crop management practices: Ecosystem-based integrated nutrients (soil) and pests (diseases, insect-pest and weed) management approaches such as conservation agriculture, alterations in cropping patterns and rotations, crop diversification, mulch cropping, cover cropping, organic agriculture, irrigation management and land fragmentation among others. Crop improvement practices: ✓ increased access to high-quality seeds/planting materials of adapted varieties, ✓ closing of yield gap in developing and least developed countries through rapid integration of new genomic technologies and ✓ development of improved site-specific crop varieties
Precipitation	Heavy precipitation events increasing in frequency over most areas.	Damage to crops; soil erosion; inability to cultivate land owing to waterlogging of soils.	very likely	
	Drought-affected area increases.	Land degradation and soil erosion; lower yields from crop damage and failure; loss of arable land.	Likely	
Air	Intense tropical cyclone activity increases.	Damage to crops; change in the normal life cycle of pests and diseases, changes in the spatial distribution of weeds such as *Striga hermonthicaı* in cereals.	Likely	
	CO_2_-induced warming resulted in rising of sea levels (excludes tsunamis).	Salinization of ground water in coastal area, estuaries, and freshwater systems; loss of arable land due to inundation of low-lying area.	Likely	

## 4 Toolbox for inclusion of climate-smart traits

Despite considerable advancement during the last decades, in terms of genomic tools and services, and marker utilization *per se*, still there is a low level of integration of molecular markers, in plant breeding programs. Several reasons could explain this, particularly in Africa, namely,

- Establishment of genotyping facilities, accessible to all breeding programs is costly
- Lack of physical infrastructure such as uninterrupted power supply, centre for data storage, among others.
- Issues related to availability of qualified, cost-effective human resources
- High cost of genotyping due to underutilization of genotyping facilities
- Inability to deliver high-quality genotyping data within a short period to meet breeding decision timelines
- Lack of comprehensive understanding of the potential of molecular markers in breeding programs
- Low or no funding for crop improvement from governments

**Figure 3:**
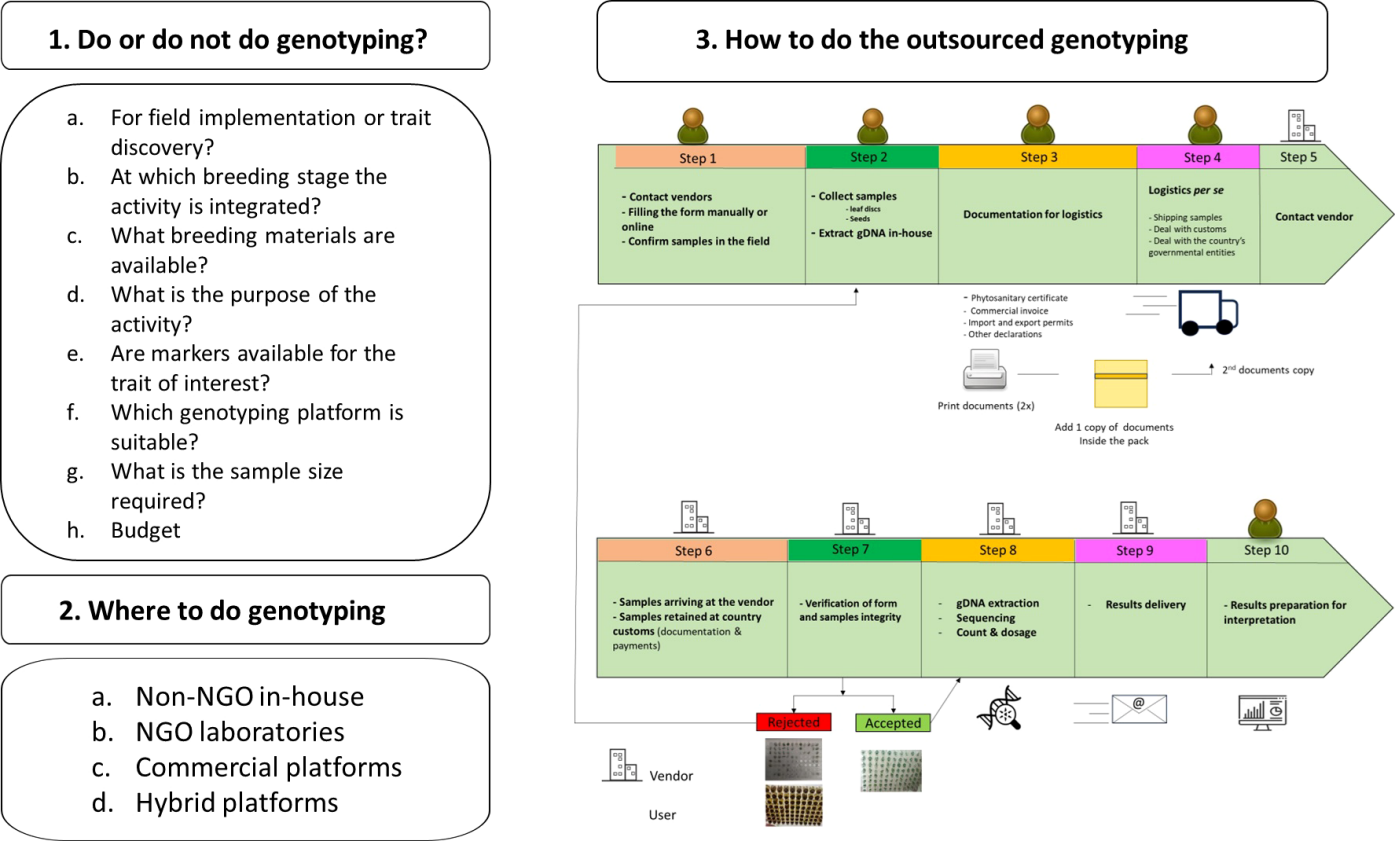
Example of workflow assessment for genotyping implementation in a breeding program (BP) as a part of a routine process in the breeding operations. Starting by defining the ideotype including trait target values, followed by accessing if genotyping is feasible, followed to understand where to perform the activity and the steps involved at the general level when outsourcing of genotyping is involved. Logistics and phytosanitary regulations are two major bottlenecks, with each country having different regulations for the same process.

To foster the integration of genomic data tools in breeding programs there must be a change in mindsets. This will require us to provide genotypic information consistently at a high-standard pro prompt and comprehensive interpretation, to assist in the implementation of advanced techniques such as marker-aided selection and genomic prediction of breeding values for further use in selection. It is very important to implement rigorous quality assurance processes that use Quality Control (QC) molecular markers for data integrity be maintained. Identifying more efficient methods of genotyping which will help in reducing the costs related with data generation and analysis. This will ensure that genotypic data is available within the breeding program workflow in time for decision-making. Additionally, it is important not to forget about embracing technological advancements in such areas as the utilization of drone-based imaging sensors and genetic algorithms based on artificial intelligence, for high-performance phenotyping since these breakthroughs can remarkably improve efficiency and precision of BPs. Concerning the implementation of genomic technologies, several questions should be posed before starting using genomic technology, including how the efficient and effective application of genomic technology can be leveraged to support BPs. Hence, there are three questions (3Qs) to address in planning a breeding program (**Figure 3**).

Advanced technologies such as Kompetitive Allele-Specific PCR (KASP) markers, high-throughput genotyping, double haploid (DH) technology, mutational signature markers, and clustered regularly interspaced palindromic repeat (CRISPR)/CRISPR-associated endonuclease 9 (Cas9) technology must serve the purpose and be applicable in a breeding program. However, certain conditions must be met for this to occur. These prerequisites involve the availability of reliable markers linked to specific traits, the access to mutational signature markers, as well the presence of advanced laboratory infrastructure and equipment conducive to the efficient utilization of CRISPR technology.

### 4.1 Publicly available marker panel platforms

With the advances in technology, particularly deep sequencing, the lowering of cost allowed the validation of other molecular panels to the crop community (**Table 2**). Several publicly available molecular marker platforms for plants include Gramene, Plant Markers Database (PMDB), and LegumeSSRdb. Gramene offers the access to SNP and SSR markers (https://www.gramene.org/; accessed November 3, 2024), whereas PMDB provides pathway-based markers for 82 plant genomes, including maize, rice, and sorghum (http://ppmdb.easyomics.org/; Mokhtar et al., 2021). In its turn, LegumeSSRdb is a comprehensive resource for SSRs in thirteen legume species (http://bioinfo.usu.edu/legumeSSRdb/; Duhan and Kaundal, 2021). Another straightforward example is the One CGIAR-CIMMYT marker platform. Initiated by the CGIAR Breeding Platform (https://excellenceinbreeding.org/toolbox/tools/kasp-low-density-genotyping-platform). This platform toolbox gathers several sets of useful markers developed for different traits and quality control (QC) in numerous crops to assess and ensure the quality and purity of breeding materials through sharing arrangements with dozens of scientists. Further, to validate the trait-specific KASP-based markers, the platform also includes the names of validated plant mid-density panels using Diversity Array Technology tag (DArTtag). Besides the above mentioned molecular marker types, there was a subsequent surge in the availability of similar platforms including the *Bean Improvement Cooperative* (http://www.bic.uprm.edu/?page_id=91) for common beans, the OZ Sorghum (https://aussorgm.org.au/sorghum-projects/) for description of quantitative trait loci (QTL) and providing resources for further marker sequence design, as well Primer3 (https://junli.netlify.app/apps/design-primers-with-primer3/) for free KASP markers design. A more recent, but equally interesting platform, is the Flex-Seq panels from Biosearch™ Technologies (https://www.biosearchtech.com/flex-seq-industry-standard-pre-designed-panels-8262) which allows different marker densities, and after made up of a standard panel for a crop any number of markers (sub-panels) can be used according to the needs of the breeding programs. Examples of these panels include maize, potato and soybean.

**Table 2:** Comparative overview of the most common PCR based DNA markers in plants.

	RAPD	AFLP	SSR	SNP
Inheritance	Dominant	Dominant/Co-dominant	Co-dominant	Co-dominant
Genomic abundance	High	High	Medium to high	Very High
DNA quantity required	Low	Medium	Low	Low
DNA quality	Lower than AFLP	Higher than SSR	Lower than SNP	High
No. of polymorphic loci	1.5-50	20-100	1.0-3.0	Thousands
Reproducibility	Low	High	High	High
Accuracy	Very Low	Medium	High	Very High
Development effort	Very Low	Low	High	High
Development cost	Low	Medium	High	Low
Running cost	Low	Medium	Low	Low
Automation	High	Low	High	High

RAPD, Random Amplification of Polymorphic DNA; AFLP, Amplified Fragment Length Polymorphism; SSR, microsatellite or Simple Sequence Repeat; SNP, Single Nucleotide Polymorphism. The number of polymorphic loci refers to the total count of loci that are polymorphic.

### 4.2 Process to incorporate molecular markers into BP workflow

**Figure 4:**
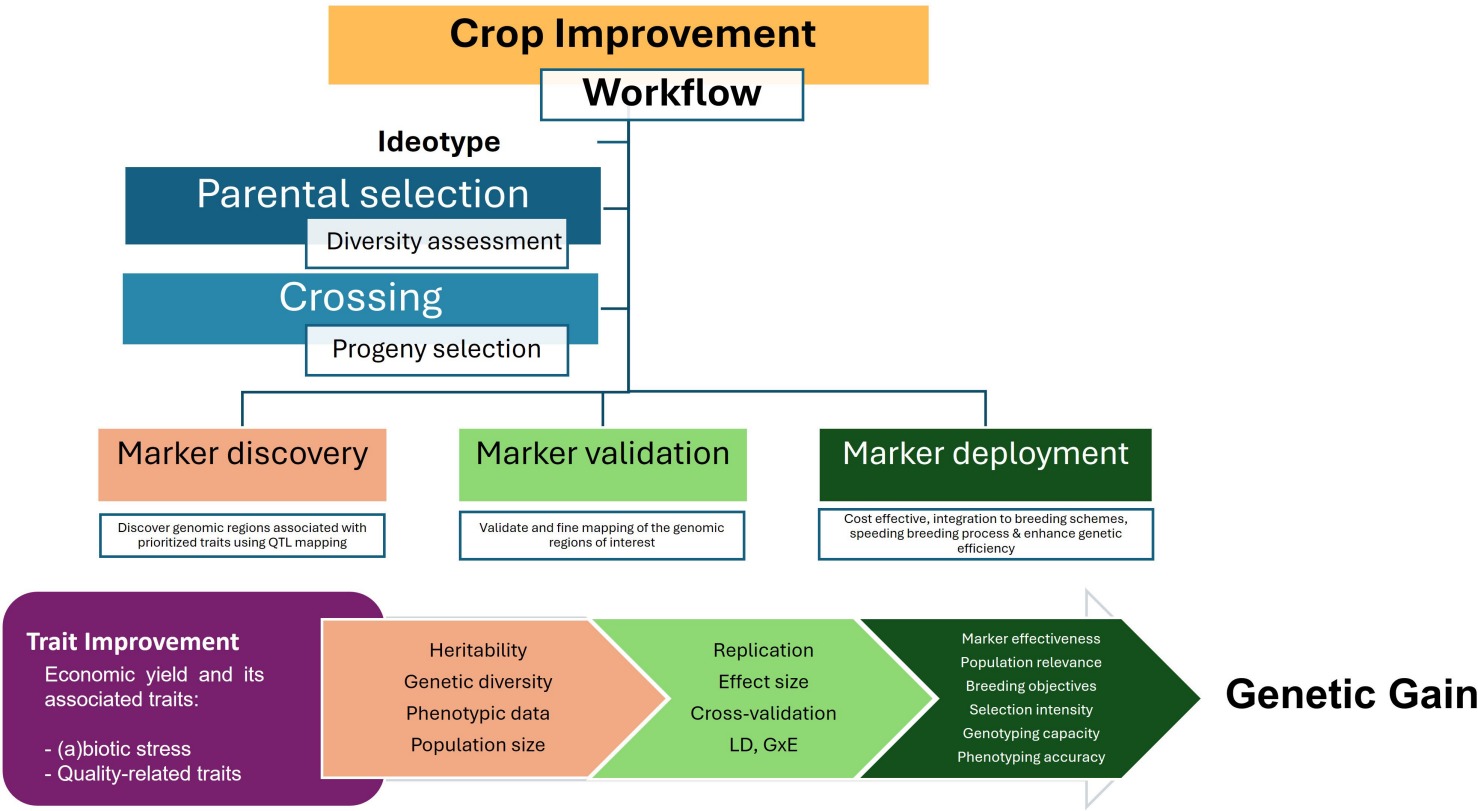
Process to incorporate molecular markers into BP workflow. The discovery, validation and deployment of molecular markers is applicable across crop plant species.

The basic steps of crop improvement include parental selection and crossing where there is a need for continuous genetic variation assessment and progeny selection. Without this yearly assessment, breeding programs cannot assure the suitability of their materials for trait improvement. Steps to integrate molecular markers include marker discovery, validation, and development (**Figure 4**) and many are the successful examples on how to apply them in BPs through diverse partnerships (Garcia-Oliveira et al., 2021, 2022, 2024).

In marker discovery, the genomic regions affecting the trait of interest for improvement are discovered through QTL mapping, including QTL identification and genome-associated mapping (GWAS), or by using reverse genetics. Once the relevant loci are revealed, they need to be validated in a wider range of targeted populations and optimized for technical efficiency. The marker validation process may involve the fine mapping of relevant loci. Finally, the validated markers are deployed in a cost-effective manner and integrated directly into the targeted breeding programs to enhance efficiency of genetic gain for specific trait.

The University of Minnesota’s (UoM, St. Paul, Minnesota, USA) Wheat Improvement Program uses markers in three main ways: 1) Parental genotyping to facilitate crossing decisions, 2) Enrichment of 3-way crosses for favourable marker alleles prior to generation advancement, and 3) Marker-assisted line purification during advanced breeding stages. Crossing parents are screened with KASP markers for approximately 50 genes influencing agronomic, end-use quality and disease-resistance related traits. Since parental genotypes are known, 3-way cross progeny can be screened and enriched for favourable alleles of segregating markers. In marker-assisted line purification, following generation advancement, lines are planted in plots of four head-rows, observed phenotypically, and screened with around a dozen KASP markers to select the most uniform and typical row to carry forward as the line. Markers are also used to screen bulks of large-scale seed increases, and if segregating markers are found, individual rows within the plot are screened to facilitate the grouping of impurities. In the case of marker-assisted line purification, special care is taken to precisely follow best experimental practices due to the importance of accurately identifying segregating rows. DNA is carefully normalized, and control genotypes for each allele, as well as negative controls, are included in each KASP experiment to facilitate accurate clustering (*personal communication Dr. Emily Conley, UoM, Minnesota, USA*).

In the breeding program of the International Potato Center (CIP, Lima, Perú), for the vegetatively propagated potato, DArTag markers (Endelman et al., 2024) are being used for genomic selection (GS), diversity analysis, and selection based on trait markers. KASP markers are used for traits with no available SNP markers in the panel array, as well to monitor the genetic identity of plants used in crossing plants (Kante et al., 2021). In Yam at the International Institute of Tropical Agriculture (IITA, Ibadan, Nigeria) KASP SNP markers are being used to distinguish different yam species (*Diascorea alata*, *D. rotundata*, *D. praehensilis*, *D. cayenensis* and *D. abyssinica*, and also tagged markers with the economically important traits such as plant vigour, sex distinguishing, flowering intensity, yam mosaic virus, among others (Agre et al., 2023). For marker discovery, other platforms with higher marker density can be of use.

## 5 Identifying trait-associated haplotypes for improved breeding

### 5.1 KASP-haplotype and epiallele underlying quantitative traits 

In breeding, trait-associated haplotype identification is valuable in the context of genetic selection through the manipulation of population allele diversity. The inclusion of haplotype information in genomic selection models can enhance the accuracy of predicting an individual’s breeding value (Sallam et al., 2020). Haplotypes not only capture more extensive genetic variation but also provide a finer resolution of genomic variation compared to individual SNPs alone. KASP-haplotype genotyping allows the detection of multiple SNPs within a haplotype, thereby improving linkage disequilibrium between causal loci and marker haplotypes and leading to more precise estimates of marker effects for genomic selection.

However, to date, from QTL mapping, thousands of loci have been described for numerous traits, many of which control only a small proportion of the trait variance. Not only is the true proportion of phenotypic variance these KASP loci explain unknown but also these haplotypes of interest change from breeding cycle to breeding cycle due to recombination and introduction of new germplasm. The use of KASP-haplotypes can capture rare genetic variants that might not be detected or accurately imputed using individual SNP markers. Additionally, in the majority simply inherited traits, haplotypes are easy to find and quantify their contribution to the trait phenotype. Yet, the genetic complexity of the target trait, the genetic diversity of the population, the trait architecture, and the availability of genomic data, all factor into the ease of identifying and quantifying haplotypes of interest. A similar ‘in-house made’ method to KASP methodology is the “Amplifluor-like” method, and competitive against KASP methodology. A primary advantage of the Amplifluor-like method lies in its unparalleled flexibility, allowing for the modification or rearrangement of all its elements. This includes the ability to alter the structure and design of allele-specific primers and universal probes, as well as the amplification program, including parameters such as temperature and duration for each step (Khassanova et al., 2023). With the availability of knock-off mastermixes, that can be cheaper, and the primers ordered from whoever provides the cheapest oligo synthesis service in a particular region, one that has already an equipped and expert laboratory, such one at the UoM can design allele-specific assays (Allele 1, Allele 2 and reverse primer), add the hex tail on one primer and a fam tail on the other, and use PACE mastermix, which is commercially available but for about half the price of KASP (https://3crbio.com/). However, in breeding programs where laboratory facilities and consumables purchase are not competitive, using of shared services available is ideal.

**Figure 5:**
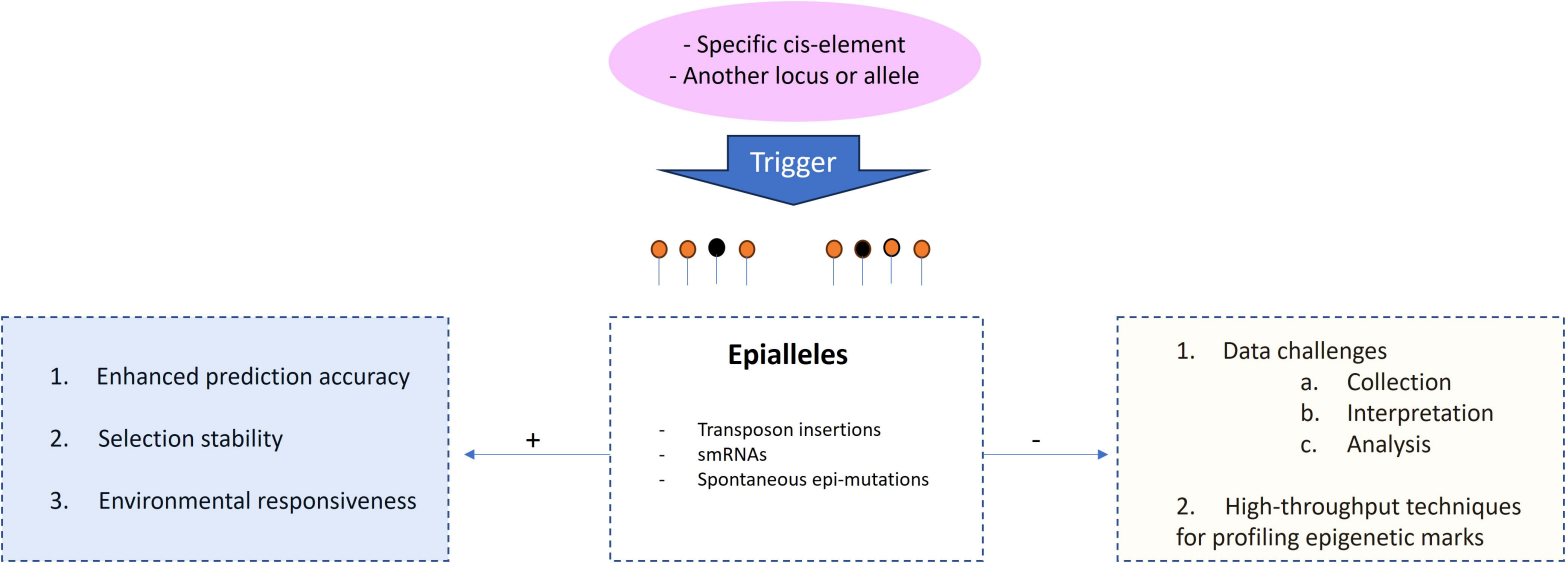
Relevance of epialleles to genomic selection. 1. Including epigenetic information, such as DNA methylation patterns associated with quantitative traits, can provide additional information beyond the DNA sequence itself, 2. Epigenetic modifications can contribute to phenotypic stability across generations by providing an additional layer of heritable variation that is less affected by short-term environmental fluctuations. When considering epialleles associated with stable phenotypes, genomic selection can facilitate the breeding of more resilient and adaptable individuals. 3. Epigenetic modifications are sensitive to environmental cues and can mediate phenotypic plasticity. Incorporating information about epigenetic marks that respond to specific environmental conditions, genomic selection models can account for genotype-environment interactions and improve prediction accuracy in varying environments. Despite continuous technological advance, careful experimental design and computational analyses are still required to extract meaningful information for selection purposes, as in the case of DNA methylation sequencing. The signals (+) and (-) refers to advantages and drawbacks, respectively.

Another motive to consider is the use of epialleles, as these can play a great role in the regulation and contribution to observed variation of quantitative traits (Gahlaut et al., 2020). Epigenetic modifications can influence gene expression levels, developmental processes, and responses to environmental cues, thereby impacting complex traits. Understanding the epigenetic regulation not only provides valuable insights into potential avenues for breeding, but also, hypothetically, and in the context of genomic selection, genomic selection models can potentially improve the accuracy of predicting breeding values, and therefore, enhancing selection efficiency, particularly at the level of environmental responsiveness and phenotypic plasticity (**Figure 5**). This approach may require a different type of marker platform entirely to get genome-wide coverage of SNPs in methylated as well as unmethylated regions. Current technologies could prove challenging, as most current high-throughput platforms tend to target unmethylated (presumably gene-rich) regions. Nevertheless, the incorporation of epialleles into genomic selection is still evolving, with the need to research effective methods for integrating these elements while understanding the complex interactions between epigenetics, quantitative traits, and breeding outcomes. Therefore, it is an exciting avenue for further improvement in breeding strategies.

### 5.2 Challenges when utilizing KASP markers in polyploid crops

An effective KASP SNP result is achieved when the marker demonstrates clear differentiation between homozygous and heterozygous alleles in the genotyping output. However, not all the results are straightforward, often leading to complex outcomes where alleles cannot be assigned to specific allele groups. Also, in polyploid crops, information on allele dosage might not always be available. In such instances, a process of SNP recalling becomes necessary, which in some cases is a challenging task that demands technical expertise and is often carried out manually, consuming significant amounts of time and the need for knowledge expertise. Beyond assessing homozygosity/heterozygosity, KASP genotyping data can be used to predict target observable traits (phenotypes) associated with specific genetic variations and based on known associations between certain alleles or SNP markers and phenotypic traits of interest, established through genetic studies and BPs.

Another matter of importance is marker data imputation, either in diploid or polyploid crops, to fill in the missing or incomplete genetic information in a dataset. It involves predicting or estimating the allelic variants (genotypes) of individuals at specific physical positions in their DNA sequence where data is missing. Genotypic data can be incomplete for various reasons, such as genotyping errors, low sequencing coverage, or missing samples. This practice is particularly valuable to enhance statistical power and to improve the ability to identify genetic associations with traits. Common imputation tools and software packages used in genomics research include IMPUTE (Marchini et al., 2007), BEAGLE (Browning and Browning, 2009), LD-kNNi (Money et al., 2015) and Minimac (Howie et al., 2012). It is not uncommon to use the imputation replacing missing data with the average dosage for a specific site and population, noting that the accuracy of imputed genotypes depends on the quality and size of the reference panel and the specific imputation algorithm used.

It is important to acknowledge the challenges associated with obtaining accurate dosage information when employing KASP markers for QC purposes. These challenges encompass the optimization of assay conditions, including primer concentrations, annealing temperatures, and PCR conditions. Additionally, primer design and specificity must be carefully considered to prevent cross-reactivity with other genomic regions, which could potentially yield false results. The quality and quantity of DNA are crucial factors, as contaminants, degradation, or insufficient DNA concentrations may compromise the reliability of dosage information. Sample variability poses another challenge, emphasizing the necessity to confirm that the chosen marker is suitable for the specific genetic background under investigation. The absence of reliable reference materials or standards for use as control samples can further complicate dosage determination. The presence of PCR inhibitors in the DNA sample, if not addressed, can impact the efficiency of the reaction. Moreover, the calibration of equipment is paramount, as inaccuracies in temperature control and fluorescence detection can lead to unreliable results. The analysis of KASP data requires robust software capable of accurately calling genotypes and dosage information. Finally, incorporating replicates and positive/negative controls in experimental setups is essential for assessing assay reproducibility and detecting potential issues.

On the quality side, and to enhance reliability, the use of control genotypes is recommended. Yet, achieving marker stability is vital, given that the same marker may yield different calls across various plates, even when considering the same sample repetition. This could be due to poor DNA quality, or in the case of polyploids, failure to normalize DNA samples to a consistent concentration. It could also be due to a low level of true residual variation at some loci following generation advancement and line purification. Consistent marker performance over time is also a crucial characteristic of high-quality KASP markers. This necessitates the identification and selection of markers that amplify well and cluster cleanly, especially in the context of defining a limited set of QC markers, as demonstrated in the case of soybeans (Chander et al., 2021).

## 6 Precision Plant Breeding with application of genotyping technologies

### 6.1 Mutation breeding in genomic era

DNA mutations are the basis of all heritable variation in organisms, providing the raw material for natural selection and evolution. However, such naturally stable mutations are relatively rare in eukaryotes compared with prokaryotes, partly due to the presence of complex DNA repair mechanisms in the former. In plants, mutation rates vary across different regions of the genome, with lower frequencies observed in areas under strong functional constraints (Quiroz et al., 2023). Specifically, the mutation rates are reduced by 50% within gene bodies and by two-thirds within essential genes, as compared to non-essential genes (Monroe et al., 2022). This reduction is likely due to the elevated activity of targeted DNA repair mechanisms in these regions. A deeper understanding of mechanisms that promote specific DNA repair could help achieve critical goals in crop improvement programs through targeted mutagenesis. The application of mutation techniques by plant breeders has a long history, dating back to 1920’s when X-ray induced mutagenesis was first realized on fruit fly (*Drosophila melanogaster*) and cereal species (maize and barley) by Muller (1928) and Stadler (1928a, b), respectively. Muller’s groundbreaking research revealed that genes typically exhibit a mutation rate ranging from 10^-5^ to 10^-6^ per locus per generation. The advent of radiation-induced mutagenesis allowed scientists to achieve higher mutation rates than those occurring spontaneously leading its application in inducing novel genetic variability through radiation in plants. Over the past century, various physical agents like X- and gamma-rays, UV light radiations, as well as particle radiations including alpha- and beta-particles, neutrons, and protons, along with chemical substances such as alkylating agents (e.g., EMS - ethyl methanesulphonate, NEU - nitrosoethyl urea, MNU - N-methyl N-nitrosourea), colchicine, EI (ethylene imine), and sodium azide (NaZ), among others, have been extensively employed. These tools have not only been instrumental for studying gene function and DNA repair mechanisms but have also been pivotal in generating novel cultivars in various crops (Penna and Jain, 2023). Globally over 3400 cultivars derived from approximately 228 plant species have been developed in 75 countries to date, utilizing a range of mutagens (http://mvd.iaea.org accessed in January 2024). Among these, radiation-induced mutagenesis, particularly with gamma rays, remains one of the most prevalent methods.

More recently, the application of accelerated particles including heavy ions (C-, H-, and Ar- ions) or protons, has gained importance for developing novel cultivars with desired traits. These particles induce mutagenesis with high biological effectiveness at low radiation doses minimizing impacts on other phenotypes (Tanaka et al., 2010; Abe et al., 2015). The precision and specificity of accelerated particles along with their energy, reduce off-target mutagenesis making them a powerful tool in plant breeding. Similarly, space breeding conducted in environments beyond Earth’s atmosphere offers another promising avenue for creating novel mutants. This approach takes advantage of two unique factors: exposure to cosmic rays and microgravity (Liang et al., 2009; Zhao et al., 2010; Liu et al., 2021a).

In any of mutagenesis methods, it is crucial to consider the direct inheritance of the DNA damage caused by the mutagen as well as the persistence of the genetically induced alterations. This phenomenon, known as mutagen-induced genomic instability can occur in the progenies that have undergone irradiation (Ma et al., 2021). Compared to accelerated particles, X-rays induce a greater extent of DNA damage and offer fewer opportunities for repair by DNA polymerases. When DNA polymerases are less efficient at correcting errors, the mutation rate increases, particularly in the case of double-strand break (DSB) mutations. This can lead to chromosomal re-arrangements and larger deletions (Pastwa et al., 2003; Tsuruoka et al., 2008; Hirano et al., 2015). High-energy particles have been used to induce qualitative traits (sterility in fruit trees and flower appearance such as colour and shape in flowers), and quantitative agronomic traits in cereals and vegetable crops (Abe et al., 2012; Kato et al., 2016; Yamatani et al., 2018; Nishio et al., 2024). The new traits obtained through these methods are useful for gene function mining, gene mapping and the development of elite alleles. Besides seed-propagated crops, *in vitro* mutation breeding in vegetatively propagated crops has also resulted in lines exhibiting several desirable traits (Suprasanna et al., 2015; Maurya et al., 2022; Zhang et al., 2024). However, there is still much to be improve in this field of mutagenesis, including optimising physical radiation parameters such as dose and high linear energy transfer (LET), understanding the impact of different irradiated particles to decrease mutation randomness as well the integrating genomic and phenomics tools to enhance mutation breeding efficiency (Nielen et al., 2018).

### 6.2 Next-Generation sequencing platforms

The establishment of next-generation sequencing (NGS) platforms in the recent advances of technology has enhanced our capacity to sequence and map crop genomes, identify causal mutations and study gene regulation. However, it should be noted that the majority of these genomes are complex and characterized by a relatively high proportion of repetitive sequences and transposons. For larger genomes, the short sequence reads (<700bp) generated by second-generation sequencing platforms like Illumina, Roche, Solid, and others may not be ideal. The second-generation sequencing can be extremely useful but requires methods to reduce complexity and target informative regions. For example, Illumina NovaSeq platform can be used for routine genomic prediction in a breeding workflow, as it is low cost, despite requiring specialized knowledge to prepare libraries, access to core facilities with the sequencing instruments, and bioinformatics expertise to process the data. In cases where this is not possible long-read sequencing such as Oxford Nanopore and PacBio, also understood as the third-generation sequencing generation platforms, need to be used. These long-read sequencing platforms offer the capability to sequence longer fragments, enabling more comprehensive coverage of large genomes, especially in highly variable genomic regions which may facilitate the identification of epigenetic marks such as DNA methylation and gene expression analysis. However, these third-generation platforms are more expensive and generally less available than those of the second-generation. These are useful for discovery-type research, whereas second-generation would be more useful for routine breeding applications. For the detection of rare point mutations in plant genomes the use of NGS still presents challenges. As an alternative, the Simple, Multiplexed, PCR-based bar-coding of DNA (SiMsen-seq) system is an opportunity for selective mutation detection using sequencing, as it detects variants at or below 0.1% frequency with low DNA input (Ståhlberg et al., 2017).

**Figure 6:**
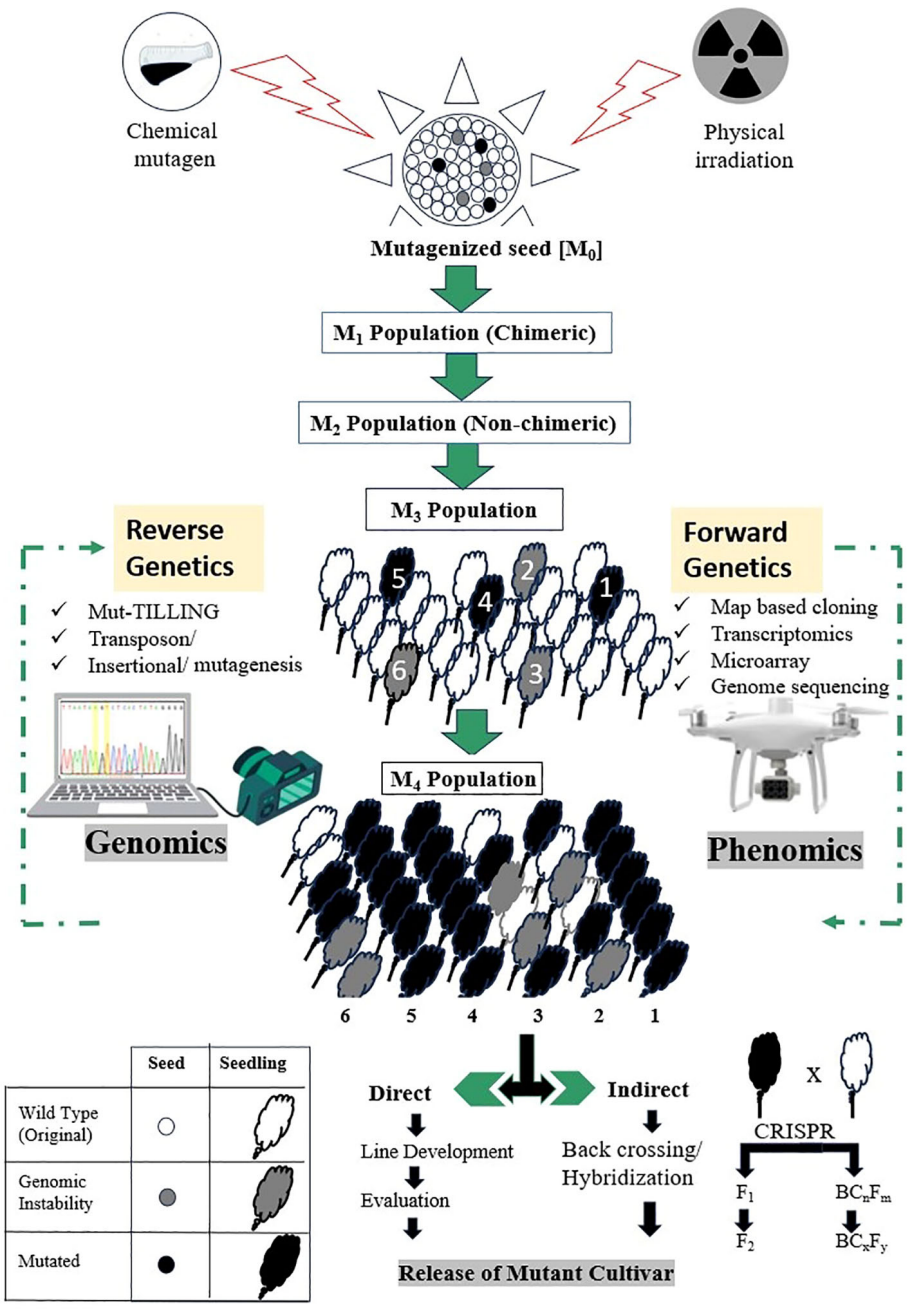
Use of high throughput genotyping and phenotyping for the development of desirable cultivars in plant mutation breeding. In forward genetics, after applying mutagenesis (such as radiation) and selecting M1 seeds, performance of precise phenotyping is done at the M2 generation. By the time the M3 generation is formed, a more refined phenotypic evaluations can be done, as the M3 plants are typically more stable and can provide clearer insights into the genetic basis of the traits of interest. This approach helps in identifying and confirming the relationship between specific mutations and the observed phenotypes. In reverse genetics, the focus is more on specific genes and their functions rather than broadly phenotyping entire populations like in forward genetics, therefore, CRISPR and gene editing can be used initially to create mutations in specific genes or may induce mutations through radiation. This is followed by phenotypic evaluation for change, starting at M2, and where the effects of mutations can be observed. By M3 generation, stable mutants can be characterised to confirm the function of targeted genes based on the phenotypes observed.

Generally, mutation breeding operates outside of the typical regulatory controls. To make breeding more efficient, molecular markers can be used in selecting materials for specific traits of interest, which are then further identified in the field. Afterwards, characterization of these mutants can be carried out using advanced NGS pipelines. More importantly, it should be noted that genome editing technologies can play a significant role in trait pyramiding by enabling the introduction of multiple site mutations for different desired plant characteristics (**Figure 6**).

### 6.3 Genomic tools for utilization of microRNAs and their native targets

MicroRNAs (miRNAs) are a type of short (19-24 nucleotides in length) non-coding RNA molecules which are present within the inter and intragenic regions of genomic DNA. Since the discovery of first miRNA *lin-4* in the nematode (*Caenorhabditis elegans*) about 30 years ago, much evidence has shown that miRNAs are ubiquitously present in eukaryotic genomes that regulate gene expression, mainly through sequence-specific cleavage and/or translation inhibition of the target mRNAs during or after transcription (Lee et al., 1993; Wightman et al., 1993; Voinnet, 2009; Sanei and Chen, 2015). As a result, miRNAs are emerging as master modulators of gene expression that could be utilized as next generation genomic tools for improvement of traits of interest in agronomically/economically important plants (Djami-Tchatchou et al., 2017; Tang and Chu, 2017; Raza et al., 2023). Most of functionally validated miRNA families are not only evolutionarily conserved among plant species, but also tend to have conserved targets which make more convenient of miRNAs for application in trait improvement in crop plants (Tang and Chu, 2017). Various types of miRNAs, including conserved miRNAs that facilitate comparative studies across species, tissue-specific miRNAs that inform on developmental processes or stress responses, and stress-responsive miRNAs that are upregulated or downregulated in response to stresses, have the ability to enhance their utility in breeding stress-resistant varieties. To date, the utilization of natural genetic variation in molecular marker-aided breeding mainly relied on either random DNA markers or so-called functional markers that derived from protein coding genes.

The continuous progress in DNA marker technology replaced previous PCR-based genotyping methods. High-throughput sequence-based single-nucleotide polymorphism (SNP) markers emerged as an attractive option because of low genotyping error rate, and amenability to automation, thereby resulting in a drastic reduction in cost per data point (Chander et al., 2021). SNPs in miRNA (MIR) genes and their target genes are widespread and can influence miRNA biogenesis and function by altering transcriptional patterns or miRNA-target interactions (Todesco et al., 2012; Liu et al., 2014). In rice, nucleotide polymorphism (GG/AA) in the terminal loop region of the osa-miR2923a precursor could be differentiated from japonica/indica cultivars which are also found to be significantly associated with grain length characteristic (Wang et al., 2013). Similarly, the naturally occurring nucleotide variation in OsmiR156h, exhibited reduced plant height, enhanced lodging resistance, increased tiller numbers per plant, and resulting in an increased grain yield (Zhao et al., 2015). Few recent examples, and exemplifying their outcome importance, include the miRNAs OsmiR168 and OSmiR395, targeting the Ago1 and ATP sulfurylase (*OsAPS1*) genes which enhanced the resistance and broad-spectrum resistance to rice blast fungus (*Magnaporthe oryzae* L.) and both pathovars *Xanthomonas oryzae* pv.oryzae (Xoo) and *X. oryzae* pv. oryzicola (Xoc), causing bacterial blight and leaf streak diseases in rice, respectively (Wang et al., 2021; Yang et al., 2022). Similarly, in alfalfa miR156 target combined stress modulators. The miR156 not only was found to module flooding tolerance by regulating physiological processes and SnRK1 gene expression (Feyissa et al., 2021) but also to influence heat, cold and drought tolerances by downregulating the SPL gene (Arshad et al., 2017a, b; Arshad and Hannoufa, 2022; Yadav et al., 2024). Therefore, the integration of NGS information together with various bioinformatics tools and transcriptomic studies may aid in the identification of the functional role of natural or induced variations at miRNAs and their target loci that could be utilized to develop molecular markers. For instance, in the case where the target gene has a desirable effect on the trait of interest but MIR serving as a negative regulator, selection of natural or induced miRNA-resistant target gene mutant strategy can be used. Contrarily, if a candidate MIR gene serve as a positive regulator and its native target gene has an undesirable effect on the trait of interest, the natural or induced MIR variant, such as the suggested isoMIR, can be utilized (Morin et al., 2008). It should be noted that compared to canonical miRNAs (https://www.mirbase.org/), their isoMIRs have often shown differential expressions, higher binding capacity, and efficiency in target cleavage in *Arabidopsis thaliana* (Jeong et al., 2013; Ahmed et al., 2014).

### 6.4 Genome editing tools for crop improvement

Using high-throughput sequencing (HTS), hundreds of miRNAs have been identified and annotated in different plants species (https://www.mirbase.org/), but their biological function still unknown. Like the study of protein-coding genes, following the identification of miRNAs-targets, their functional characterization is necessary by the creation of gain-of-function or loss-of-function analyses. To perform gain-of-function studies, miRNA genes or miRNA-resistant target genes could be overexpressed using various techniques such as traditional constitutive promoter, full-length cDNA of miRNA genes, or the two-hit artificial miRNA vector system, which is highly adaptable to plant transformation techniques (Teotia et al., 2020). As a result, most of miRNA studies have been conducted on Arabidopsis due to the ease of the floral-dip transformation method (Zhang and Unver, 2018).

While RNA interference (RNAi) and site-directed genome engineering techniques such as zinc finger nucleases (ZFNs) and transcription activator-like effector nucleases (TALENs) approaches were successfully employed for loss-of-function studies (Curtin et al., 2011; Li et al., 2012; Ainley et al., 2013; Liang et al., 2014). Compared to traditional genetic mutants of protein-coding genes, miRNA mutants are less effective due to the small size of miRNAs and have several members with overlapping functions. Thus, the simultaneous knockdown of all miRNAs in a family can be the best way to confirm the biological function of a specific miRNA family. To fill this lacune, different molecular techniques that knockdown of miRNAs by target decoys/mimics such as target mimics (TMs), short tandem target mimics (STTMs), molecular sponges (SPs) and artificial miRNAs (amiRNAs), have been demonstrated to be useful to underpin the role of these miRNAs. For instance, Franco-Zorrilla et al. (2007) identified an endogenous long non-protein coding gene INDUCED BY PHOSPHATE STARVATION 1 (IPS1) which altered the protein levels of PHOSPHATE2 (PHO2) by modulating the effects of miR399 in Arabidopsis.

It was observed that both IPS1 and PHO2 had highly conserved sequence motifs that contain complementary binding site for the phosphate (Pi) starvation–induced miRNA miR399. However, IPS1 showed three additional nucleotides bases, which provoke central mismatches in the miR399 binding site by forming of central “bulge” opposite the expected miRNA cleavage site, thus avoiding miR399-guided cleavage but instead sequesters of miR399. This endogenous regulatory mechanism of miRNA activity was termed as endogenous “target mimicry” or eTM which is commonly referred to as miRNA decoys/mimics technology. Based on the strategy of mimicking target transcripts, new methods to inhibit miRNAs, particularly Short Tandem Target MIMICs (STTMs)-based knockouts, have been developed in miRNA functional studies (Tang et al., 2012; Teotia et al., 2016). STTMs contain two miRNA-binding sites, which not only efficiently silence some highly abundant miRNAs but also can be used to study the interactions between two miRNAs by inserting two different miRNAs in the same STTM construct (Zhang et al., 2017; Peng et al., 2019).

In the area of *in silico* miRNA target prediction in plants, numerous bioinformatics tools can be used as web servers and freely accessed including TAPIR (https://ptarpmir.cu-bic.ca/), TarHunter (http://www.biosequencing.cn/TarHunter/), psRNATarget (https://www.zhaolab.org/psRNATarget/), psRobot (http://omicslab.genetics.ac.cn/psRobot/) and WPMIAS (https://cbi.njau.edu.cn/WPMIAS/), these three last lacking miRNA-initiated phasis information (Liu et al., 2021b). Furthermore, PHASIS (https://github.com/atulkakrana/PHASIS) and PhaseTank (https://phasetank.sourceforge.net/) were developed to support phasiRNAs prediction in plants. The database sRNAanno is presented as a comprehensive collection of phasiRNA loci in plants (http://plantsrnas.org/), but still, does not indicate which phasiRNA sites are triggered by miRNAs. More recently, TarDB (http://www.biosequencing.cn/TarDB/) surged as a web resource for exploring relatively high-confidence miRNA targets and miRNA-triggered phasiRNAs in plants RNA information (Liu et al., 2021b). An example of such specific miRNA targeting use can be exemplified by the recent 216 drought-responsive identified miRNAs (DRMs) based on 28 drought stress-specific sRNA datasets using the RiceMetaSys: Drought-miR database (Kumar et al., 2024). At a genome-wide scale, the high throughput degradome/PARE (Parallel analysis of RNA ends) sequencing techniques have enabled the characterization of miRNA cleavage sites. Computational pipelines such as CleaveLand, PARESnip and sPARTA can help further analyse the data sets originated through degradome/PARE-seq (Addo-Quaye et al., 2009; Kakrana et al., 2014; Folkes et al., 2012). Databases for plant miRNA such as miRBase (https://mirbase.org/) and PmiREN (https://www.pmiren.com/) can be utilised not only to archive and annotate miRNAs but also as a miRNA encyclopaedia with function and resource tools.

### 6.5 Precision gene editing tools for crop improvement

Over the past three decades, the unconditional success of genetically modified (GM) crops allowed the way for harnessing novel plant breeding technologies in crop improvement. Despite the widespread adoption of GM crops, the presence of foreign genes in them since their development has been central concern of public acceptance and potential biosafety issues in many parts of the world (Kumar et al., 2020). These concerns have driven the development of new plant breeding techniques, such as gene editing, which enable precise modification of plant genomes without introducing foreign DNA (Wang and Doudna, 2023).

The foundation of gene editing in plant was established with the discovery of I-SceI (meganuclease) induced double stranded breaks in a site-specific manner that enhanced homologous recombination in plants (Puchta et al., 1993). Meganucleases, also referred to as homing endonucleases, are sequence-specific endonucleases which found in prokaryotes, archaea, and unicellular eukaryotes. These endonucleases are often small proteins that are encoded by mobile genetic elements, either introns or inteins, and cleave relatively long sequences of DNA (ranging from 12-40 bp) resulting in higher specificity and lower off target cutting (Stoddard, 2014). The primary challenge facing meganuclease technology is the significant engineering efforts required to create enzymes with novel DNA specificity, particularly due to the overlapping nature of DNA-cleavage and DNA-binding domains in these homing endonucleases. Despite this hurdle, engineered meganucleases have demonstrated some success in various gene editing functions in plant species (Daboussi et al., 2015); however, the production of modified homing endonucleases is both time-consuming and lacks the necessary flexibility.

To address the problem associated with meganucleases, another class of site-specific nucleases (SSN) such as zinc finger nucleases (ZFNs) were developed by fusing an artificial DNA-binding domain (DBD) of a versatile class of eukaryotic transcription factors – zinc finger proteins (ZFPs) to the non-specific DNA cleavage domain of type II restriction endonuclease *FokI* (Kim et al., 1996). This achievement spawned from the discovery that the DNA-binding domain and the cleavage domain of the *FokI* function independently of each other (Li et al., 1992). The FokI nuclease operates as a dimer, thus necessitating the design of two ZFN molecules to target a single site. Each ZFN molecule binds itself to opposite strands of DNA molecule, causing a double-strand break. One notable development is that they have modified a vast variety of genomes in plants (Urnov et al., 2010). Yet, the only thing standing in the way is its challenges such as off-target cutting, and cytotoxicity which led to the development of a new class of nucleases: transcription activator-like effector nucleases (TALENs). TALENs, akin to ZFNs, arise from the fusion of catalytic domain of the FokI endonuclease with a DNA-binding domain called transcription activator-like effectors (TALEs), derived from the protein produced by plant pathogenic *Xanthomonas* spp. Bacterium (Christian et al., 2010; Bogdavone and Voytas, 2011).

Until a few years ago, both ZFNs and TALEN were the most powerful programmable site-specific technologies for genome engineering. These techniques are modular in form and function, comprised of DNA-binding domain fused to *FokI* cleavage domain that recognize target sequences by protein motifs and which requires the assemblance of specific proteins to each target, therefore having low specificity in recognizing and cleave the DNA targets (Gaj et al., 2016). Recently, clustered regularly interspaced short palindromic repeats/CRISPR-associated nuclease 9 (CRISPR/Cas9) techniques have been proved to be useful to genome editing because CRIPSR/Cas system has the advantage of recognizing targeted genomic sites by base complementarity between the single-stranded guide RNA (ssgRNA) and the target DNA. So far, CRISPR/Cas9 has been applied to introduce specific genetic modifications aimed at improving desirable traits in cereals, vegetables (including root and tuber crops) and fruit trees (Osakabe and Osakabe, 2015; Rodriguez-Leal et al., 2017; Jaganathan et al., 2018; Tripathi et al., 2020; Biswas et al., 2021; Ma et al., 2023).

Nonetheless, it is important to note that CRISPR/Cas is not a universal tool, as it has limitations related to the short sequence length and the specificity of the microRNA (MIR) genes. These limitations affect the design of guide RNAs (gRNAs) targeting MIR genes and their applicability, respectively. Precise genome editing requires accurate gene sequences and knowledge of their functions, which is facilitated by the whole-genome sequencing data currently being generated. Transgenic plants overexpressing cleavage-resistant targets such as miR164-resistant *SlNAM2* (Hendelman et al., 2013) in tomato, and miR139-resistant *OsTCP21* (Zhang et al., 2016a) and miR166-resistant *OsHB4* (Zhang et al., 2018a) in rice, have been created and used to investigate miRNAs functions. Among the various loss-of-function systems available, miRNA decoy techniques [Target Mimic (TM) and Short Tandem Target Mimic (STTM)] and the CRISPR/Cas9 genome-editing system are the most utilized. As an example, the resistance to powdery mildew in barley accessions and mutant types was shown to be controlled by the *mlo* gene (the wild allele is *Mlo*) (Jørgensen, 1992). The loss of function of *mlo* mutants confers a durable and broad-spectrum resistance to powdery mildew. Yet, the *mlo*-associated resistance brings along growth and yield penalties, where the expression of *Mlo* in *mlo* genotypes is sufficient to confer single-cell susceptibility. Pleiotropic effects may explain this phenomenon, and many breeding programs may need to address it. In polyploid crops, this technique may require additional backcrossing cycles to eliminate any unwanted mutations (**Figure 6**).

In hexaploid wheat, the TALEN-generated Tamlo-R32 mutant targets the three wheat *MLO1* genes (*TaMLO-A1*, *TaMLO-B1* and *TaMLO-D1*) and is characterised by a 300kb pair targeted deletion in the *MLO-B1* locus that retains crop growth and yields while conferring resistance to powdery mildew. Through epigenetic changes, this mutant exhibits a significant upregulation of *TaTMT3B* (Tonoplast monosaccharide transporter 3) locus transcript expression. The use of the CRISPR/Cas9 tool demonstrated that this genetic arrangement counteracts the negative effects associated with *mlo* mutations while maintaining strong disease resistance in wheat (Li et al., 2022).

In strawberry, *fvesep3* mutated using CRISPR/Cas9 has produced the desirable trait of parthenocarpic fruits, which is highly sought in strawberry breeding programs (Liu et al., 2020b; Pi et al., 2021). Similarly, in polyploid and parthenocarpic bananas, where asexual breeding methods are less efficient, the utilization of CRISPR/Cas9 systems can enhance mutation efficiency and introduce traits of economic importance such as reduced plant height (Shao et al., 2020), improved quality (Kaur et al., 2020; Awasthi et al., 2022), and host plant resistance to pathogens (Tripathi et al., 2021).

Recently, and in response to some constraints presented by Cas9, Zheng et al. (2024) demonstrated in rice that CRISPR Cas12a is a more efficient tool compared to its Cas9 counterpart for generating knockout mutants of a miRNA gene. With this improvement, it seems possible to achieve editing efficiencies of up to 100%. Due to its ability to create larger deletions, which facilitates the generation of loss-of-function mutants of targeted genes, this new system appears better suited for developing genome-wide miRNA mutant libraries, at least in rice.

Improving crops by pyramiding of a few desired genes in a single elite line have been facilitated by marker-assisted selection (MAS). As shown for diverse crop species in the following sections, CRISPR take this a step further by the so-called multiplexing of a number of genes that have been mutated by CRISPR/cas in a single event. In fact, a mutant collection can be generated by use of CRISPR as demonstrated for maize in the BREEDIT pipeline by Lorenzo et al. (2023) where a first attempt of multiplexing 48 growth-related genes resulted in more than thousand mutant lines which can be screened for improvement in complex traits such as drought and heat tolerance.

### 6.6 Case studies

#### 6.6.1 Rice

In rice, noteworthy examples of precision editing include the knockout of three seed weight-related genes, namely GW2, GW5, and TGW6, which lead to an increase in grain weight (Xu et al., 2016). In this crop, the targeted disruption of the promoter region of the SWEET gene has been shown to enhance rice’s resistance to bacterial blight (Oliva et al., 2019). In quest to boost African rice’s agronomic potential, a method known as multiplex CRISPR/Cas9 was used to target *HTD1*, *GS3*, *GW2*, and *GN1A* loci, which bear domestication genes for the cultivation of rice. It has been established that mutations in these genes can lead to significant increase in seed output when tested in landraces of rice (Lacchini et al., 2020). In a different experiment, the *GS3* locus was targeted alongside with the *qSH1*, *An-1*, and *SD1* loci, that all together led to rapid domestication of wild allotetraploid rice (Yu et al., 2021). These examples highlight the effectiveness of multiplex CRISPR/Cas9 technique in rice that can lead to improve and advance crop domestication efforts.

#### 6.6.2 Wheat

In wheat, CRISPR/Cas-mediated genome editing had been targeted traits such as host plant resistance to powdery mildew (*TaMLO* & *TaEDR1*) (Shan et al., 2013; Wang et al., 2014; Zhang et al., 2017), seed yield (*TaGW2* & *TaGASR7*) (Zhang et al., 2016, 2018b; Wang et al., 2018a), fusarium head blight resistance (*TaHRC*) (Su et al., 2019), herbicide resistance (*TaALS* & *TaACC*) (Zhang et al., 2019a, 2019), quality traits such as low gliadin (*VIT2*), starch and amylose (*TaSBElla*) contents (Jouanin et al., 2019; Liu et al., 2020; Li et al., 2020b), high haploid induction rate (*TaCENH3α*) (Lv et al., 2020), male sterility (*TaMs1 and TaNP1*) (Okada et al., 2019; Li et al., 2020c), increase of P uptake (*TaPHO2-A1)* (Ouyang et al., 2016) and storability (*TaLOX2*) (Shan et al., 2014; Zhang et al., 2016). These modifications were performed by using edits such as knockout, multiplexing, base editing, primer editing and HDR replicon (Li et al., 2021). In addition, the knockout of numerous conserved domains in approximately 100 α-gliadin family members facilitated the development of a low-gluten cultivar suitable for individuals with celiac disease (Sánchez-León et al., 2018). The existence of three sub-genomes (A, B, and D) in wheat makes the application of multiplex genome editing beneficial, although achieving precise editing remains challenging as current efforts predominantly focus on random mutations and knockouts through non-homologous end joining (NHEJ) repairing. In crops like wheat NHEJ is commonly exploited to induce these random changes in the DNA sequence, allowing researchers to disrupt or deactivate targeted genes for various purposes.

#### 6.6.3 Solanum genus

In tomato (*Solanum lycopersicum* L.), the multiplexing of the coding regions of *SELF-PRUNING* and *SELF-PRUNING 5G*, together with cis-regulatory regions of *CLV3* and *WUS* or open reading frames (ORFs) of GGP1, allowed the generation of tomato fruits with compact plant architecture, synchronized fruit ripening, day length insensitivity, enlarged fruit size and increased vitamin C levels (Rodriguez-Leal et al., 2017; Li et al., 2018). Additionally, leveraging the multiplex capacity of CRISPR/Cas9, researchers successfully targeted five different genes associated with lycopene content accumulation in tomatoes (Li et al., 2018). More recently, in potatoes (*Solanum tuberosum* L.), the functional knockouts of several *S*-genes, namely *StDND1, StCHL1*, and *DMG400000582* (*StDDMR6-1*), lead to the generation of tetraploid potatoes with increased resistance against late blight (Kieu et al., 2021). The mutation of *DMR6* homologues indicates that *StDMR6-1* and *StDMR6-2* have two different biological functions, with the first involved in pathogen resistance whereas the second involved in plant growth. It was observed that *Stdnd1* and *Stchl1* mutants reduced infection lesion sites, whereas StDMR6-1 not only reduced the infection lesion sites but also the percentage of infected leaves. These results are very promising for the potato breeding since pathogen races in potatoes rapidly overcome the existing plant resistance.

#### 6.6.4 Seed production

CRISPR/Cas9 offers a potential solution to avoid the need for farmers to yearly seed purchase due to the phenomenon of heterosis. Heterosis, also known as hybrid vigour, is only maintained within the F_1_ generation, which means that the production of these superior offspring is laborious, costly, and often unaffordable to small scale farmers. Nevertheless, if CRISPR/Cas9 is used then it can help to overcome such limitations and allowing for the development of stable and improved seed cultivars that retain desirable traits across generations. This would mean that farmers would have a reducing dependence on purchasing new seeds every year, and, therefore, providing a more sustainable and cost-effective solution for farmers.

Through simultaneous editing of *REC8*, *PAIR1*, *OSD1*, and *MTL* genes, researchers have been able to fix the favourable F_1_ traits (Wang et al., 2019). When *REC8*, *PAIR1*, and *OSD1* genes were knocked down simultaneously, and meiosis was replaced by mitosis, it was enabled the production of asexual hybrid rice seeds and the preservation of the hybrid vigour (Khanday et al., 2019). In strawberries, and other fruits crops, the knockdown of all the six anthocyanin transport genes *RAP* (*REDUCED ANTHOCYANINS IN PETIOLES*) using the CRISPR/CAS9 system has produced white octoploid fruits instead of the red-coloured typical berries (Gao et al., 2020). This demonstrates the simultaneous knockout of homoeologous alleles as a tool to breed polyploid plants.

Similarly, the generation of DH (double-haploid) homozygous lines is a labour-intensive and expensive endeavour that needs laboratory infrastructure and has variable efficiency. The timeline for the whole DH line production process takes from several months to a few years depending on factors such as the species involved, the haploid induction success rate and the chromosome doubling technique efficiency. However, targeted genome editing-mediated haploid induction has provided a more rapid option with lines being generated within one year (Hooghvorst and Nogués, 2020). Several key genes have been targeted for this purpose including *MATL* (*MATRILINEAL*), *CENH3* (*CENTROMERE-SPECIFIC HISTONE 3*) and *DMP* (*Domain Membrane Proteins*) genes (Zhu et al., 2020; Kuppu et al., 2020; Zhong et al., 2019a). In wheat CRISPR/Cas9- mediated targeting of the *MATL* gene led to an inheritance rate of 18.9% haploid progeny (Liu et al., 2019a, b) while targeting maize *DMP* gene resulted in maternal haploids with an efficacy range of about 0.1–0.3% (Zhong et al., 2019a, b). An improved version of this system is the HI-editing technology (HI-Edit) which makes available transgene-free edited inbred lines lacking inducer parental DNA. By eliminating the necessity for introgression, this approach minimizes the time and expense involved (Kelliher et al., 2019). As technological advancement carries on, techniques like the CRISPR-Combo system which performs simultaneous gene activation and editing using CRISPR hold a lot of promise in terms of speeding up the identification process of markers-free genome-edited lines that are highly efficient. These techniques also offer an opportunity to screen mutants at both genome and transcriptional levels (Pan et al., 2022a). As these technologies continue to evolve, it’s expected that the process of obtaining and studying gene-edited lines will become more efficient and streamlined. Hence, we can look forward to easier and faster ways of getting and understanding these edited genetic lines in the future.

## 7 CRISPR/Cas9 without DNA donor involvement

Ever since the first mention of CRISPR/Cas9 technology, back in 2013 (Li et al., 2013; Nekrasov et al., 2013; Shan et al., 2013) there have been some major improvements. In the past, this technology relied on using a DNA donor template to introduce the desired genetic changes. However, the most recent developments have allowed for the utilisation of CRISPR/Cas9 without the need for a DNA donor template, opening new possibilities for gene editing. This modified version of CRISPR/Cas9, instead of using a DNA donor, uses the Cas9 enzyme to produce targeted breaks in specific parts of the genome. These breaks then activate the cell’s natural DNA repair mechanisms, which can result in gene disruptions, insertions, or deletions. The CRISPR/Cas9 technology involves the cut of a specific sequence in the organism’s genome. This cutting action is triggered by an RNA called single-guide RNA (sgRNA), which is designed to match the target DNA sequence. Double-stranded breaks are generated when the homologous sequences of the guide RNA are in proximity to the specific protospacer adjacent motif (PAM) sequence. This allows for genetic modifications to occur during the subsequent repair mechanisms (Asmamaw and Zawdie, 2021; Singh et al., 2022). More recent advancements in CRISPR technology made it possible to combine an engineered reverse transcriptase and prime editing (PE) RNA with CRISPR/Cas9. This new approach eliminates the need for double-stranded breaks or donor templates and enable changing specific bases, deleting targeted sections, inserting, or even a combination of those. This methodology has been demonstrated on crops such as maize, lettuce, tobacco, rice, and bread wheat, and is a potential solution to tackle concerns raised by the public and regulatory bodies about CRISPR/Cas9-derived plants (Svitashev et al., 2015; Woo et al., 2015; Liang et al., 2017).

## 8 CRISPR/Cas9 prime-editors in plants

CRISPR/Cas9 prime-editors have become a powerful tool for precise editing of plant genomes. Prime-editing combines the Cas9 nuclease with an engineered reverse transcriptase and a prime editing RNA (pegRNA), which enables targeted base editing, insertions, deletions, and combinations without the need for double-stranded breaks or donor templates. Currently, the most usual editors used in the CRISPR system are the base editors, and characterised for making specific base changes, limited to A, C, G, and T. Instead, prime editors (PEs) offer a broader range of mutations, including all 12 possible transition and transversion mutations, as well as small insertions and deletions (INDELs), without the need for DNA double-strand breaks (Anzalone et al., 2019). One example of prime editing in plants is the modification of the acetyl-CoA carboxylase (*ACCase*) gene. This gene is crucial for fatty acid biosynthesis, and the loss-of-function mutations on it can lead to severe developmental abnormalities or even be lethal for plants (Baud et al., 2004). *ACCase* is also the target of many commercial herbicides. In rice, the *OsACC* gene confers herbicide resistance. Through base editing libraries, targeted mutations were introduced in both *OsACC* and acetolactate synthase (*ALS*) genes, which are targets of herbicides used for plant weed control in field crops (Garcia et al., 2017). Using this approach key amino acid residues that are related to resistance to herbicides were revealed (Liu et al., 2020; Kuang et al., 2020). In addition, various mutations in the acetyl-coenzyme A carboxylase 1(*OsACC1*) gene, have been discovered, resulting in herbicide resistance (Xu et al., 2021). These examples illustrate the significance of prime editing technology for crop improvement purposes specifically when it comes to development of herbicide-resistant cultivars and understanding the functional importance of specific substitutions of amino acids. Although its optimization is still underway, prime editing in plants promises precise genetic manipulation and therefore serves as a promising tool towards better agricultural achievements.

## 9 Opportunities and obstacles in applying CRISPR/Cas9-derived methodologies

**Figure 7:**
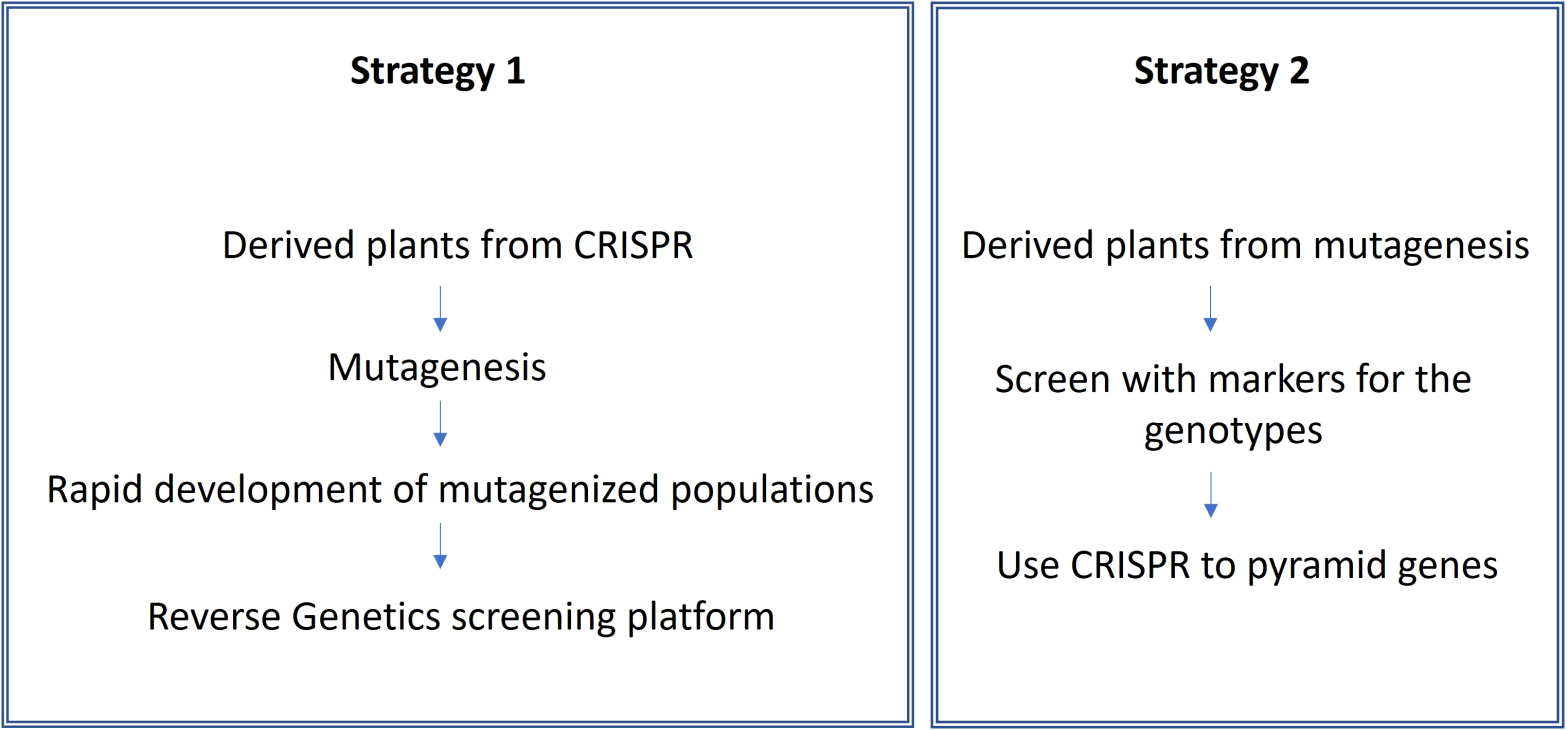
Strategies on how CRISPR and mutagenesis can be applied based on current knowledge.

The access to complete DNA sequencing information and a thorough understanding of gene functions has facilitated the use of CRISPR-Cas9 technology for precise modulation of key genes. This also allows for the quick creation of new genetic resources to improve specific traits. Nevertheless, it’s crucial to note that direct sequencing methods might miss out on certain heterozygous alleles. To overcome this limitation, methods like TILLING (Targeting Induced Local Lesions IN Genomes) can be used to identify mutations at specific loci in the modified genetic material. Furthermore, DNA markers can be used to detect mutations at the early stages of plant growth. TILLING brings several benefits in this scenario. Libraries developed through TILLING contain preserved genetic resources, usually in the form of seeds, and the genomic DNA extracted from mutated materials can remain stable for many years. This allows for the identification of specific allele groups linked to different traits, which can be safeguarded and shared with the public. However, caution is advised when targeting traits controlled by multiple genes. The current landscape provides numerous prospects for breeders and scientists, depending on their gene preferences. When dealing with stable genes, hybridization is often the preferred strategy. Conversely, if the genes of interest are dominant, transformation approaches may be the most effective option. For recessive genes, mutation methodologies are frequently utilized. These strategies, based on our existing knowledge, are implemented in plant research to tackle urgent global challenges (**Figure 7**).

By employing refined NGS pipelines (Sahu et al., 2020) to characterize mutants generated through diverse chemical and irradiation techniques, new allelic variants in the genomes of various crops can be generated. This approach potentially may establish mutation breeding as a fundamental pillar in the efforts towards nutritional and food security. It is also an opportunity for the generation, validation, and removal of deleterious alleles, since these are mainly accumulated in the pericentromeric regions of chromosomes due to less selection efficiency (Dwivedi et al., 2023). Yet, one must keep in mind that these regions house a great amount of “housekeeping genes” that could produce deleterious effects if disturbed. Additionally, these regions might be protective of these types of genes. Whereas the CRISPR process transforms and introduces non-native genes, it can also generate transgene-free derivatives through genetic segregation via self-pollination. This is achieved by providing a fluorescent cassette marker genetic segregation, which signals the presence of the CRISPR/Cas9 construct. Another approach is to introduce suicidal genes (CMS2 and BARNASE genes) into the T_0_ plants, which selectively eliminate transgene-containing pollen and embryos through the crossing. These methods are, however, still not applicable for asexually propagated crops like potato, cassava, and banana. It’s important to note that while CRISPR is often associated with simple mutations, it can also facilitate the introduction of larger foreign DNA fragments, which is categorized as NGT 2 in the EU’s soft regulation framework (EC, 2023). This distinction is important, as it highlights that CRISPR’s capabilities extend beyond short, simple mutations typically seen with chemical-induced mutations, and therefore, enabling more complex genetic modifications.

Among the known challenges, it is worth mentioning the difficulty in predicting sgRNAs efficiencies, requiring the optimization of Cas protein derivates. The current delivery systems for Cas and sgRNAs have limitations, highlighting the need for improvement in the CRISPR specificity system, and the urgent need to expedite improvements in these delivery mechanisms (**Table 3**).

**Table 3:** Challenges and options to overcome in the CRISPR/Cas9 systems delivery.

System	Challenge	Possible solutions	Reference
CRISPR/Cas9	Editing efficiency	Increase CG contents in the sgRNA by selecting sgRNA with high CG content	Ren et al. (2019)
		Use of native U6 promoters	Long et al., 2018; Zhou et al., 2018; Ren et al., 2021; Zhang et al., 2022
		Use of Cas12a/b proteins to extend CRISPR usage scope	Ming et al., 2020
		Use of fluorescent labelling	Ali et al., 2023
	Multiplex Editing	Usage of PTG/Cas9 system	Wang et al., 2018b
		Use of multiplexed tRNA-gRNA2.0 system	Pan et al., 2021
	Deliver efficiency into plant cells	Use of vector-mediated and nanoparticle delivery systems; use of cut-dip-budding delivery system in herbaceous and woody plants; use of merismatic and plant germlines	Laforest and Nadakuduti, 2022; Wang et al., 2022b; Cao et al., 2023; Ali et al., 2023
	Off-target effects	Use of engineered precision variants of Cas9, Cas12a and deaminases; use high-fidelity Cas9	Zhang et al., 2019; Jin et al., 2020a; Chen et al., 2017
	Side effects	Segregation, field evaluation	Discussed below (This work)
	Low regeneration rates lead to high chimerism	Use of adventitious regeneration protocols; use of chimeric genes	Malabarba et al., 2020; Pan et al., 2022b
	Generation of transgene-free plants	Use of lipid transfection, viral vectors, delivery of components directly as functional sgRNA and Cas9 protein	Mahmoud et al., 2022; Ma et al., 2020; Pompili et al., 2020
	Activation of gene expression	Stabilization of donor DNA and CRISPR system through the introduction of 5’-phosphorylated double-stranded oligodeoxynucleotide (dsODN)	Lu et al., 2020
	Precision	Use of base editor tool	Gao, 2021
		Improve the efficiency of the primer editor for usage	Chen et al., 2021; Nelson et al., 2022
	Epigenetic modifications	Use of more efficient epigenome editors	Wilson et al., 2020
	Use of double-haploid technology	Higher production of haploids	Zhong et al., 2019a; Kuppu et al., 2020; Gao, 2021
	Regulations	Non-integration of foreign DNA/RNA into the target site	Wang et al. (2022c)

The abundance of genomic sequence data and the need to uncover target genes associated with essential agronomic traits highlights the importance of establishing precise links between genotypes and phenotypes. Therefore, better delivery systems in CRISPR technology are needed, including reducing the dependency on tissue culture methods and to facilitate the widespread adoption of this technology in agriculture.

While the technology seems useful for specific traits such as plant diseases and pests, or quality traits; when it comes to more complex traits, such as yield, the potential of CRISPR technology may be seen with some reservations. CRISPR technology seems to be a valuable tool to facilitate trait introgression and support the mitigation of linkage drag in the case of simply inherited traits. However, in both CRISPR and induced mutation, there is the need to address challenges of specific genetic variance generation for highly quantitative traits, which is crucial to achieve the necessary increases in production. We are likely to experience great innovations in crop genetics in a multitude of ways, yet, if accurate phenotyping will not accompany this evolution, it will be challenging to observe a rapid evolution in crop improvement. Integrating precise genotyping with accurate phenotyping will significantly contribute to the effective implementation of CRISPR technology and accelerate the progress in crop improvement efforts.

Regarding regulatory frameworks, currently, there are two regulatory frameworks that several countries have adopted to classify crops developed using CRISPR and other genome editing technologies: product-based and process-based regulations (Ishii and Araki, 2017). In the product-based framework, only the final product is controlled in terms of regulation. Consequently, if there are no exogenous transgenes in the commodity, then it is considered as a non-GMO. Conversely, under a process-based approach, any crop produced through CRISPR/Cas9 technology shall be considered a genetically modified organism (GMO). The choice between these two frameworks will determine how genome-edited crops should be managed and labelled. Different countries have adopted different approaches, and specific examples of countries following each framework can be found in related literature (Chen and Gao, 2020; Singh et al., 2022). Regulatory setups significantly influence commercialization as well as public reception of CRISPR/Cas9-derived crops across various regions globally. Despite technologies such as ZFN, TALEN, Meganucleases (MN), and CRISPR can be used for specific genome editing without the incorporation of foreign DNA into the target site, it is crucial to recognize that these tools are not perfect and sometimes irregularities occur during the process. The off-target effects are one major worry because they may result in cell death or cause genomic instabilities (Lazzarotto et al., 2020) while sequencing cannot detect or differentiate between natural and genetic engineered variability. Research demonstrates that prime editors (PEs) do not induce detectable pegRNA-independent off-target edits in plants (Jin et al., 2021). Using CRISPR to treat genetic diseases in humans, side effects such as chromosomal rearrangements can occur after genome editing (Raimondi et al., 2024), besides editing a gene may turn on or off other genes associated with that gene. In plants, off-targets and side effects may be segregated out during meiosis or discarded during the multigenerational field evaluation selection because of performance and therefor side effects may be of lesser practical problem in seed propagated plants. For asexually propagated plants, field assessments may be the only way of recognizing such effects. Nevertheless, continued research in the field of genome editing is needed to address these concerns and ensure the safe and precise application of these technologies in economically important crops. For small breeders that obtain royalties for their varieties, or payment for certified seeds and farmer saved seeds, outsourcing CRISPR technology is a good option for them to get access to specific traits without investing in costly laboratory facilities and staff with specialised skills.

## 10 Connecting the dots – Fostering the integration of inclusive genomic innovations in the smallholder farmers’ agritech investments

The successful adoption and implementation of inclusive genomic innovations (IGIs) in the agriculture sector of the Global South, especially in low- and mid-income countries, continue to pose challenges. The primary obstacle stems from a lack of understanding and awareness regarding the specific needs and requirements of the target users (UNDP, 2022).

For instance, during the late 1990s, cotton crop was significantly affected by insecticide-resistant Lepidopteran pests in India resulting in excessive use of insecticides that caused not only a serious environmental and human health problem but also led to economic problems for cotton growers that contributed to farmer indebtedness. The introduction of genetically modified cotton [*Bacillus thuringiensis* (Bt) cotton] showed not only a direct positive impact on yield gains through reduced crop losses by good control of native bollworm *Helicoverpa armigera*, but indirectly also led to reductions in insecticide use (Gutierrez et al., 2020) resulting in 134% increase the income of smallholder farmers (Subramanian and Qaim, 2010). In China, the widespread planting of Bt cotton cultivars not only successfully controlled the effects of the polyphagous pest *Helicoverpa armigera* among multiple crops but also provided great advantages by providing a 10-fold increase in the products of Chinese farmers between 1996–2018 (Pray et al., 2011; Zhaozhi et al., 2022; ISAAA, 2020). Similarly, the adoption of Bt technology in other crops such as Bt brinjal contributed 22% higher revenues compared to non-Bt cultivars in Bangladesh (Shelton et al., 2020).

Despite these encouraging outcomes, public perception, concerns about potential risks and unintended consequences, and ethical considerations contribute to the ongoing debate. An alternative approach includes the use of ‘Fast Identification of Nucleotides variants by DigITal PCR’ known as FIND-IT, which offers a promising solution for rapid identification of pre-targeted genetic variants or rare alleles in large and very large populations. With this method libraries of 500,000 knockout barley mutant individuals, can be screened within only two weeks (Knudsen et al., 2021; Madsen et al., 2024). Contrary to CRISPR methodology, FIND-IT is not subjected to governmental regulations as a non-GM technique. Additionally, compared to TILLING methods, FINF-IT offers simpler technological requirements and greater sensitivity in detection. This high-throughput ddPCR method does not require transformation or tissue culture protocols making it a scalable and efficient approach for screening and targeting desired traits in crops with low mutation-density variant populations.

The innovative upfront genomic innovation (GI) technologies may not directly serve as tools for smallholder farmers in many countries but rather offer products that can benefit them. It is undeniable that smallholder farmers are a significant portion of the global agricultural workforce, operating, majority of the times, under resource constrains. Therefore, investments in high technologies such as genotyping are opportunities to improve yields, reduce production costs and even mitigate risks associated with the increased climate variability. The solution is to bridge the gap that exists between traditional farming methods and modern agricultural practices. By offering them tailored solutions to address their unique challenges, genomic innovations may make a difference. The information got from the genetic codes of crops, not only offers better cultivars that are better locally adapted but also facilitates the selection of the most desired traits and allows the farmers to produce higher-quality crops with fewer resources. Yet, to fully understand the potential of genomic innovations requires addressing issues such as access, affordability and capacity building to ensure equitable distribution of benefits across the farming communities. Besides the previous examples, in Sub-Saharan Africa, the development of drought-tolerant maize cultivars using MAS breeding techniques using genomic data (molecular markers) has helped farmers mitigate the adverse effects of climate change and improve food security (https://dtma.cimmyt.org/). The same was shown by the introduction of disease-resistant cassava cultivars in Nigeria and Uganda (https://www.nextgencassava.org/). With an exponential amount of information available, the future holds the opportunity to achieve tailored genomic technologies for smallholder farming. Such examples of this are MAS, GS and genome editing tools. One way to foster the integration of inclusive genomic innovations into agritech investments for smallholder farmers is to prioritize capacity building and knowledge transfer, including through training programs, extension services and farmer field schools.

Innovative financing mechanisms, including impact investing, venture capital and blended finance models may support resource mobilization and scale up the adoption of inclusive genomic innovations and therefore, accelerate their adoption by the farmer community. An example of this is the effort on biofortification through breeding by the HarvestPlus program (www.harcestplus.org) that over 20 years facilitated the release of more than 420 biofortified cultivars in different staple crops across Asia, Africa and Latin America (Dwivedi et al., 2023). So far, most of the biofortified cultivars developed through crossbreeding approaches, however, molecular breeding efforts using IGIs for the same purpose are gaining momentum, thus resulting in speed up the development of biofortified cultivars (Badu-Apraku et al., 2018; Sheoran et al., 2022). The molecular markers developed for biofortified traits such as pro-vitamin A, Fe and Zn content in edible parts of different staple crops could be utilized to improve locally adapted farmer-preferred cultivars in sub-Saharan Africa and Latin America.

Despite all the above-mentioned strengths in empowering smallholder farmers through genomic innovations, careful consideration must be given to potential risks including genetic erosion, biodiversity loss, and unintended environmental consequences. One must be aware that, beyond technology adoption, it is important to take into consideration that the use of CRISPR ultimately raises important concerns regarding Intellectual Property Rights (IPRs), particularly as these technologies can impact breeders’ rights, the ownership of genetically edited crops but also issues on the accessibility of the developed innovations in agricultural biotechnology. Potential solutions could involve not only the development of clear legal frameworks, encourage the open access to CRISPR technology findings to the breeders, develop fair and flexible licensing agreements so breeders can use CRISPR technology while providing compensations to innovators, improve communication and collaborations between the different stakeholders, as well promotion for fairer regulations that protect both innovators and breeders.

The future holds space not only to explore genome editing tools for unprecedented precision and efficiency but also the space to integrate the omics data technologies that promise deeper insights into gathering real-time data for real-time decision-making. A major contribution of these advanced technologies, from the breeder perspective, includes the implementation of multiple field trials, which should be emphasised for mutational and CRISPR-Cas generated parental lines after they have demonstrated success in greenhouse conditions.

To conclude, the integration of various approaches, whether it be through mutation breeding, gene editing, crossbreeding or other techniques, holds the potential for a more comprehensive and effective strategy in enhancing plant traits for increased production.

## Glossary

*AgMIP*: Agricultural Model Intercomparison and Improvement Project is a collaborative global research effort aiming to enhance agricultural models’s accuracy in predicting crop yields, food security assessment, and climate change impacts on agriculture and was established in 2010
*CRISPR*: Clustered Regularly Interspaced Short Palindromic Repeat system used to modify/edit a cultivar of plant species. It facilitates site-directed mutagenesis
*CRISPR off-target*: Accidental or unintentional alterations in different DNA regions, different from the intended spots, with similar but not exact sequences to the target site
*Crop productivity*: Efficiency of crop production, measured as yield *per* unit area. It can exceed 100% (as shown in **Figure 1** of this paper), when yields surpass previous standards (global average) due to improved varieties, better management practices, or favourable conditions
*DSB*: Double-strand break (DSB) mutations and refers to a type of genetic alteration in which both strands of the DNA molecule are severed or broken at the same location. This disruption in the DNA structure can lead to various genetic changes, including mutations, deletions, insertions, and rearrangements
*KASP™*: Kompetitive Allele-Specific PCR, is a genotyping technology used to detect single nucleotide polymorphisms (SNPs) and small insertions or deletions in DNA samples
*KASP haplotyping*: Technology that allows the detection of SNPs in DNA sequences. It is a form of competitive PCR where allele-specific primers are designed to selectively amplify target DNA sequences. KASP haplotyping specifically focuses on identifying and characterizing haplotypes. Haplotypes are sets of closely linked genetic markers that tend to be inherited together. Both KASP haplotyping and MAS contribute to genomic selection by providing genetic information, KASP haplotyping specifically focuses on haplotype structures, whereas MAS encompasses a broader range of marker-assisted techniques for selecting individuals based on genetic markers associated with traits
IGIs: Inclusive genomic Innovations
*IPRs*: Intellectual Property Rights, are legal protections granted to individuals or entities for their creations of the mind or intellect
*ISIMIP*: The Inter-Sectoral Impact Model Intercomparison Project is an international research initiative aimed at assessing climate change’s potential impacts on diverse sectors, extending beyond agriculture to encompass water resources, ecosystems, health, economics, and more
*Mutation breeding*: The use of mutagens, such as radiation or chemicals, to induce genetic mutations in plants, leading to increased genetic variability generation of new allele variation for trait improvement
*Mutational signature marker*: These are specific patterns of mutations found in DNA indicating the underlying processes or exposures that caused those mutations. These signatures can be associated with various factors, including environmental influences and intrinsic factors, and adapted from Human genetics
*NGT*: New Genomic Technologies are a variety of techniques that alter the genetic material of an organism. Those considered equivalent to conventional plants (NGT 1 plants) would be exempted from most requirements of the GMO legislation. https://www.europarl.europa.eu/news/en/agenda/briefing/2024-02-05/2/new-genomic-techniques-debate-and-vote-on-new-eu-rules
*NUE*: Nitrogen Use Efficiency is the measure of how effectively plants utilize nitrogen from fertilizers, encompassing uptake for growth and minimizing losses through processes like leaching and volatilization
*QC*: Quality control ensures that the genetic data used in research is robust, reliable, and suitable for interpretation and analysis. By implementing QC measures, breeders can verify the genetic purity of the F_1_ offspring, thereby confirming these are genuine hybrids derived from the intended parental lines. Moreover, QC in breeding programs supports the monitorization of the genetic diversity within the populations. It ensures that breeding stocks retain a broad spectrum of alleles, and therefore, reducing the risk of inbreeding depression and preserving the potential for future genetic improvement
*STTMs*: Short Tandem Target Mimic are a class of synthetic small RNAs that are designed to regulate the expression of specific genes in plants by modulating microRNA (miRNA) activity
TALENs: Transcription Activator-Like Effector Nucleases, are a type of engineered proteins used for targeted genome editing in various organisms, including plants, animals, and even human cells
TILLING: Targeting Induced Local Lesions IN Genomes, is a high-throughput method used for identifying and characterizing mutations in specific genes of interest
